# Unraveling the Metabolic‐Cardiovascular Disease Nexus: An Epidemiological Network Analysis Identifies Central Indicators

**DOI:** 10.1002/mco2.70967

**Published:** 2026-09-03

**Authors:** Xin‐yi Cai, Jia‐feng Zhang, Hao Huang, Wei Tang, Qian Lv, Yan Song, Xing‐xing Zhang, Quan‐cai Cai, Jian Zou, Yong‐quan Shi, Tuo Li

**Affiliations:** ^1^ Department of Endocrinology Changzheng Hospital Shanghai China; ^2^ Department of Endocrinology No. 905 Hospital of PLA Navy Shanghai Changzheng Hospital Shanghai China; ^3^ Department of Epidemiology Changhai Hospital Shanghai China; ^4^ Department of Statistics School of Public Health Chongqing Medical University Chongqing China

**Keywords:** cardiovascular diseases, Cardiovascular‐kidney‐metabolic syndrome, CKM, metabolic disorders, CardioMet, scoring systems, screening

## Abstract

Cardiovascular diseases (CVD) remain a leading cause of death globally, yet assessing the combined impact of multiple metabolic disorders on CVD risk is challenging due to a lack of comprehensive tools. We aimed to develop and validate a novel CVD screening model based on metabolic clustering networks. Using data from 4066 adults enrolled in the STONE study (China) through stratified sampling, we constructed a holistic network map of metabolic health, incorporating obesity, dyslipidemia, glucose disorders, and hepatic, renal, thyroid, and bone conditions. Through network analysis, 12 central and clinically accessible indicators—including abdominal obesity, fatty liver, LDL‐C, HbA_1c_, eGFR, TSH, and bone density—were selected to establish the CardioMet12 scoring system, with an AUC of 0.771 (95% CI: 0.733–0.808), sensitivity of 0.756, and specificity of 0.732. A score above 56 corresponded to an observed CVD prevalence exceeding 50% in the STONE cohort. External validation in an independent US population‐based epidemiological sample demonstrated satisfactory performance. Higher scores were significantly associated with metabolic syndrome diagnosis and advanced cardiovascular‐kidney‐metabolic staging in both cohorts. In conclusion, CardioMet12 is a robust metric that captures the complex interplay among multiple metabolic disorders, offering a comprehensive and proactive tool for enhanced CVD screening and risk evaluation.

## Graphical abstract

In the STONE cohort with 4752 Shanghai citizens, the CardioMet12 CVD scoring system was established by clustering individuals across seven metabolic dimensions. Based on analysis from Chinese and US epidemiological cohorts and network analysis, CardioMet12 emerges as a robust metric that considers the complex interplay among metabolic disorders, supporting a more proactive and comprehensive approach to CVD screening and evaluation.

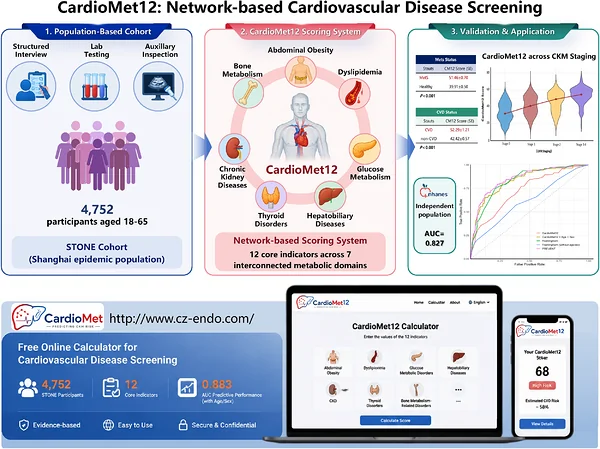

## 1 Introduction

Metabolic dysfunction plays a pivotal role in cardiovascular disease (CVD) development through shared pathophysiological mechanisms, including chronic inflammation, oxidative stress, vascular dysfunction, and insulin resistance, that converge on accelerated CVD progression and organ failure [1]. Robust metabolic health is therefore a prerequisite for pan‐vascular protection. Yet in daily practice, clinicians still rely on legacy risk engines, most prominently the Framingham Risk Score and its contemporary recalibrations (e.g., SCORE2). Although widely used, existing models may not fully capture multidimensional metabolic dysfunction and may require recalibration in some populations [2, 3].

Frameworks conceived to capture the broader metabolic milieu, such as metabolic syndrome (MetS) and, more recently, cardiovascular‐kidney‐metabolic (CKM) syndrome, underscore the intertwined biology of adiposity, dysglycemia, dyslipidemia, hypertension, and renal impairment [4, 5, 6, 7, 8]. Nevertheless, both continue to rely predominantly on categorical thresholds that compress the continuum of risk; they only partially incorporate hepatopancreatic and inflammatory axes, and several criteria still allow the diagnosis to be satisfied after downstream conditions (e.g., overt diabetes, chronic kidney disease) have already emerged, by which point opportunities for primordial prevention are narrower [9, 10, 11]. Consequently, current models strive for simplicity yet fall short of comprehensive risk stratification: conventional MetS definitions show suboptimal performance in many Asian populations [12, 13], disregard granular metabolic heterogeneity, and may call for laboratory tests that are often unavailable or unreimbursed in low‐ and middle‐income settings.

To address these shortcomings, we randomly sampled 4066 Shanghai community‐dwelling adults (18–65 years), ensuring representativeness of about 26 million residents. Using 61 low‐cost biomarkers spanning seven interconnected metabolic dimensions, we identified 12 central metabolic indicators and developed CardioMet12, which was externally validated in the National Health and Nutrition Examination Survey (NHANES).

In summary, our study delivers a practical screening tool that (i) relies exclusively on modifiable metabolic markers, (ii) integrates multisystem information, (iii) uses only inexpensive, widely available assays, and (iv) is developed in Chinese adults and independently validated in the NHANES population—thereby directly targeting the key pain points that limit current cardiometabolic risk stratification frameworks. To streamline real‐world adoption by clinicians and researchers, we have also deployed CardioMet12 as a free, browser‐based calculator (http://www.cz‐endo.com).

## 2 Results

### 2.1 Participant Characteristics

**FIGURE 1:**
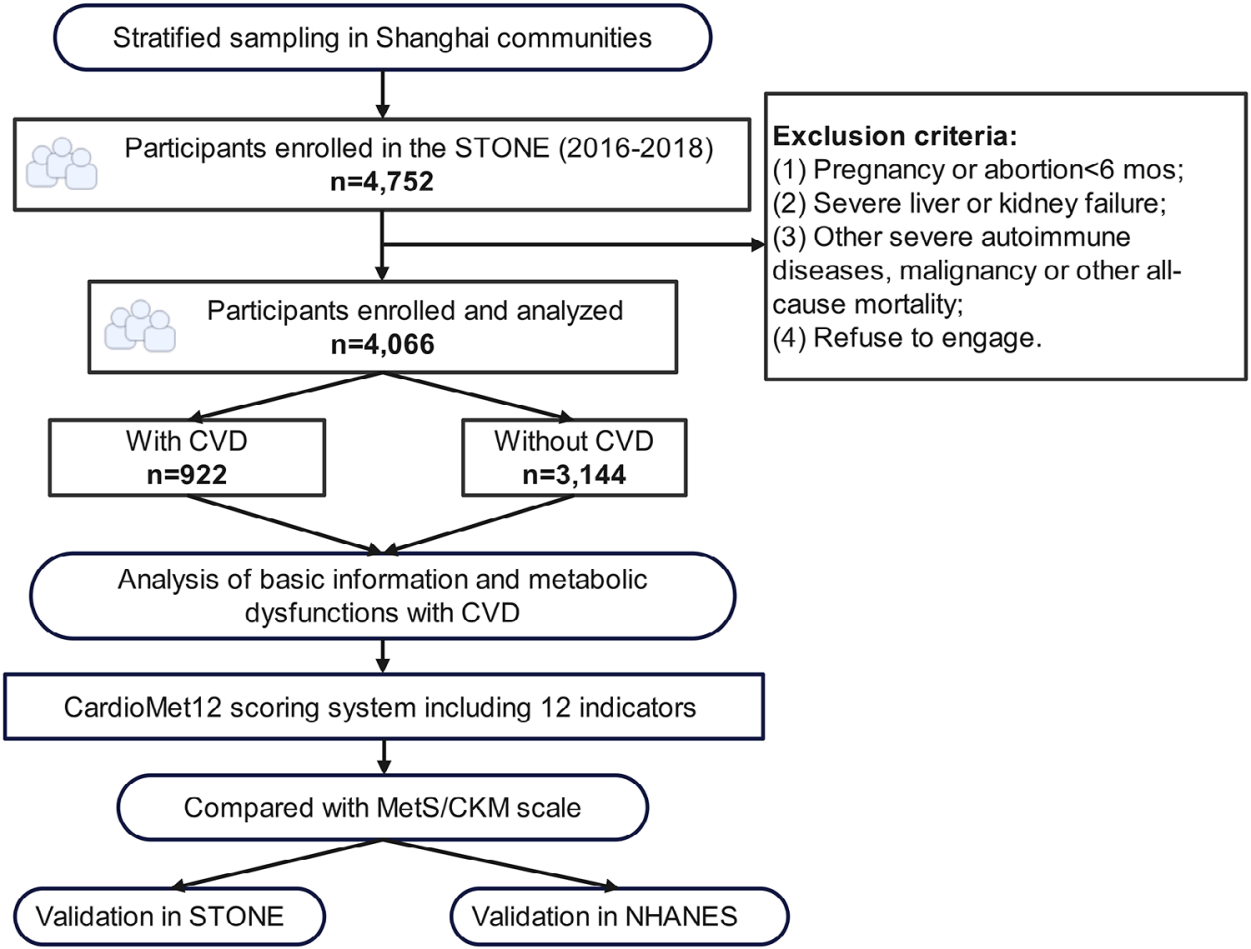
Flowchart illustrating the participant selection process, STONE. CKD, chronic kidney disease; STONE, Shanghai Thyroid autOimmuNity: an Epidemiological research; CVD, cardiovascular diseases; MetS, metabolic syndrome; CKM, cardiovascular‐kidney‐metabolic syndrome; NHANES, National Health and Nutrition Examination Survey.

The epidemiological STONE (Shanghai Thyroid autOimmuNity: an Epidemiological research) survey is a public health epidemiological research across 18 districts and 242 community health service centers in Shanghai, China. A total of 4752 participants met the entry criteria and were included in the study, representing approximately 26 million Chinese citizens. After applying the exclusion criteria, 4066 participants, aged 18–65, were included in the study, conmprsing 2291 females and 1775 males (Figure 1). Demographic and baseline characteristics of the participants are presented in (Table S1), and similar findings were reported for both sexes.

### 2.2 Correlation Analysis of Basic Information and Cardiovascular Diseases

The associations of demographic characteristics, lifestyle behaviors, and mental health with CVD were first examined. Significant associations with CVD were observed for age and sex and CVD (both *p *< 0.001). Among individuals with CVD, 51% were male, compared to 42% of those without CVD, and the average age was 55 years for those with CVD versus 37 years for those without.

Initial analyses revealed differences in educational attainment, household composition, employment status, place of birth, and number of children between individuals with and without CVD (*p *< 0.001) (Table S1). However, after adjusting for age, these associations were no longer significant, indicating that age was a confounding factor.

Lifestyle behaviors were also analyzed [14]. After adjusting for age, significant associations persisted between CVD and contraceptive history (*p *< 0.001), smoking status (*p *< 0.001), and alcohol consumption (*p* = 0.003), demonstrating the continued impact of these factors on CVD prevalence.

### 2.3 Metabolic Interaction Network and Its Relation With CVD

**FIGURE 2:**
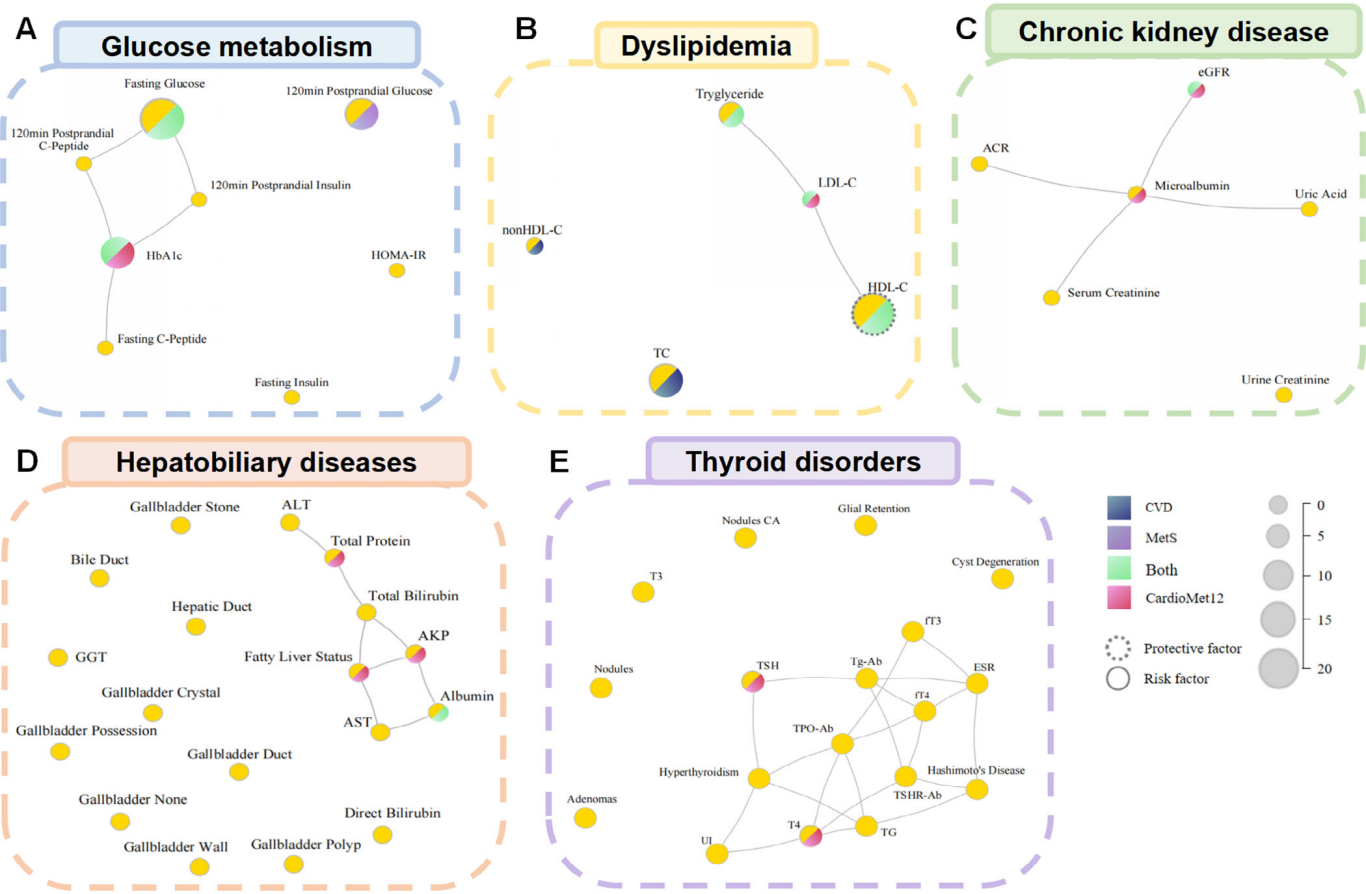
Network‐based assessment of CVD risk indices. Network analysis of indicators among glucose metabolism (A), dyslipidemia (B), chronic kidney disease (C), hepatobiliary diseases (D), and thyroid disorders (E). HbA_1c_, glycosylated hemoglobin A1c; HOMA‐IR, Homeostatic Model Assessment of Insulin Resistance; HDL‐C, high‐density lipoprotein cholesterol; LDL‐C, low‐density lipoprotein cholesterol; TC, total cholesterol; TG, triglyceride; non‐HDL‐C, non‐HDL cholesterol; eGFR, estimated glomerular filtration rate; ACR, albumin‐to‐creatinine ratio; ALT, alanine aminotransferase; AST, aspartate aminotransferase; AKP, alkaline phosphatase; GGT, gamma‐glutamyl transpeptidase; T_3_, triiodothyronine; T_4_, thyroxine; fT_3_, free triiodothyronine; fT4, free tetraiodothyronine; TSH, thyrotropin; Tg‐Ab, thyroglobulin antibody; TPO‐Ab, antithyroperoxidase antibody; TSHR‐Ab, thyrotropin receptor antibody; ESR, erythrocyte sedimentation rate; UI, urinary iodine.

Seven different metabolic‐related diseases were considered: obesity, dyslipidemia, glucose metabolic disorders, hepatobiliary diseases, thyroid disorders, chronic kidney disease (CKD), and bone metabolism‐related disorders [15]. The clinical indices are listed in Table S2. To assess the relationship between each index and CVD, a correlation analysis was performed, and a network‐based approach was employed to construct the networks of clinical indices and identify central indicators within each metabolic health target to cardiovascular outcomes (Figure 2 and Table 1). Under FDR‐corrected thresholding (*q* < 0.05), differential‐change network densities ranged from 0.000 to 0.267, with corresponding sparsities of 1.000 to 0.733 (Table 2). Betweenness centrality rankings were highly concordant between FDR and unadjusted thresholds: four of six domains (obesity, lipid, glucose, CKD) yielded identical top three rankings, and the remaining two (hepatobiliary system and thyroid) shared the same top three variable sets with only internal reordering, confirming robust indicator prioritization.

**TABLE 1:** Characteristics and profiles of metabolic health included in CardioMet12, stratified by the presence or absence of cardiovascular diseases.

Characteristic	CVD (*n* = 922^a^)	Control (*n* = 3144^a^)	*p* ^b^
Male	468 (51%)	1,307 (42%)	<0.001
Age (years)	55 (50, 59)	37 (29, 46)	<0.001
Missing	5	31	
BMI (kg/m^2^)	24.7 (22.8, 26.9)	23.3 (21.2, 25.8)	<0.001
Missing	0	3	
Abdominal obesity	507 (55%)	1,238 (39%)	<0.001
SBP (mmHg)	136 (124, 150)	125 (115, 136)	<0.001
Missing	0	3	
DBP (mmHg)	84 (77, 92)	79 (72, 86)	<0.001
Missing	0	4	
Liver function			
Total protein (g/L)	77.6 (74.8, 80.5)	78.4 (75.6, 81.4)	<0.001
Missing	41	160	
TBil (µmol/L)	10.0 (7.7, 12.9)	9.9 (7.6, 13.0)	0.6
Missing	37	158	
FLD	433 (47%)	915 (29%)	<0.001
AKP (mIU/L)	77 (65, 91)	67 (55, 81)	<0.001
Missing	51	166	
Kidney function			
eGFR (mL/min/1.73 m^2^)	102 (95, 107)	113 (104, 120)	<0.001
Missing	41	162	
Microalbuminuria (g/L)	4 (3, 9)	4 (3, 9)	0.3
Missing	51	248	
Glucose metabolism			
HbA1c (%)	5.60 (5.30, 5.90)	5.30 (5.10, 5.50)	<0.001
Missing	58	165	
FPG (mmol/L)	5.47 (5.10, 5.94)	5.17 (4.81, 5.57)	<0.001
Missing	71	185	
Lipid profile			
TG (mmol/L)	1.35 (0.93, 1.96)	1.06 (0.75, 1.57)	<0.001
Missing	65	191	
HDL‐C (mmol/L)	1.41 (1.15, 1.71)	1.51 (1.25, 1.81)	<0.001
Missing	37	148	
LDL‐C (mmol/L)	3.33 (2.83, 3.94)	3.02 (2.51, 3.59)	<0.001
Missing	70	177	
Thyroid function			
TSH (mIU/L)	2.38 (1.72, 3.31)	2.28 (1.63, 3.20)	0.031
Missing	37	149	
T4 (nmol/L)	102 (90, 113)	101 (91, 112)	0.5
Missing	37	147	
Bone density			<0.001
Normal	364 (55%)	1286 (68%)	
Bone loss	238 (36%)	534 (28%)	
Osteoporosis	54 (8.2%)	79 (4.2%)	
Missing	266	1245	

Abbreviations: AKP, alkaline phosphatase; BMI, body mass index; DBP, diastolic blood pressure; eGFR, estimated glomerular filtration; FLD, fatty liver disease; FPG, fasting plasma glucose; HbA1c, glycated hemoglobin; HDL‐C, high‐density lipoprotein cholesterol; LDL‐C, low‐density lipoprotein cholesterol; SBP, systolic blood pressure; T4, thyroxine; TBil, total bilirubin; TG, triglyceride; TSH, thyrotropin.

^a^ Categorical variables are presented as count with percentages [*n* (%)]. Continuous variables are presented as median with 25th–75th centiles [Median (Q1, Q3)].

^b^ Pearson's chi‐squared test; Wilcoxon rank sum test.

**TABLE 2:** Network metrics across metabolic domains.

Domain	Network	Edge count	Possible edges	Density	Sparsity
Obesity	CVD absent (control)	10	10	1.000	0.000
Obesity	CVD present (case)	10	10	1.000	0.000
Obesity	Differential change network	0	10	0.000	1.000
Lipid metabolism	CVD absent (control)	9	10	0.900	0.100
Lipid metabolism	CVD present (case)	9	10	0.900	0.100
Lipid metabolism	Differential change network	2	10	0.200	0.800
Glucose metabolism	CVD absent (control)	28	28	1.000	0.000
Glucose metabolism	CVD present (case)	23	28	0.821	0.179
Glucose metabolism	Differential change network	5	28	0.179	0.821
Liver and gallbladder	CVD absent (control)	53	153	0.346	0.654
Liver and gallbladder	CVD present (case)	24	153	0.157	0.843
Liver and gallbladder	Differential change network	33	153	0.216	0.784
Thyroid	CVD absent (control)	55	153	0.359	0.641
Thyroid	CVD present (case)	33	153	0.216	0.784
Thyroid	Differential change network	34	153	0.222	0.778
CKD	CVD absent (control)	8	15	0.533	0.467
CKD	CVD present (case)	10	15	0.667	0.333
CKD	Differential change network	4	15	0.267	0.733

The analysis revealed a significant association between obesity and CVD. Body mass index (BMI), waist circumference (WC), and abdominal obesity demonstrated significant relationships with CVD (*p *< 0.001). Age‐adjusted logistic regression confirmed that abdominal obesity (*p *= 0.047), BMI (*p *< 0.001), and WC (*p *< 0.001) remained robustly correlated with CVD. These results are consistent with the literature, which highlights that abdominal obesity poses a higher vascular risk compared to general obesity, even among individuals with a normal BMI range of 18.5–25 kg/m^2^ [16, 17]. Consequently, abdominal obesity was selected as the primary measure for the downstream analysis.

The lipid profile was then investigated. Initial analyses revealed significant correlations between total cholesterol (TC), triglycerides (TG), high‐density lipoprotein cholesterol (HDL‐C), low‐density lipoprotein cholesterol (LDL‐C), and non‐HDL‐C with CVD (all *p *< 0.001). After age adjustment, TG (*p* = 0.029), HDL‐C (*p *< 0.001), LDL‐C (*p* = 0.052), and non‐HDL‐C (*p* = 0.017) nearly maintained significance. Network analysis identified LDL‐C as a central node (betweenness = 1, *p* = 0.020). Given its central role in the present analysis and CVD pathophysiology, LDL‐C was identified as the key indicator for lipid profiling [18, 19, 20, 21].

For glucose metabolism, significant correlations with CVD were found for fasting plasma glucose (FPG), 2‐h plasma glucose (2hPG), fasting C‐peptide, 2‐h postprandial C‐peptide, glycated hemoglobin A_1c_ (HbA_1c_), Homeostatic Model Assessment of Insulin Resistance (HOMA‐IR), hyperglycemia (*p *< 0.001), and 2‐h postprandial insulin (*p* = 0.017). Age‐adjusted models confirmed significant associations for FPG, 2hPG, fasting insulin, 2‐h postprandial insulin, fasting C‐peptide, HbA_1c_, and HOMA‐IR (*p *< 0.05). Network analysis identified HbA_1c_ as the key indicator (betweenness = 3.5, *p* = 0.032), consistent with its established role in blood glucose regulation [22]. Chronic hyperglycemic state, represented by elevated HbA_1c_, promotes the development of arteriosclerosis [23, 24, 25]. Therefore, HbA_1c_ was identified as a key indicator for glucose metabolism in relation to CVD.

For hepatobiliary diseases, significant correlations with CVD were observed for albumin (Alb), γ‐glutamyl transferase (GGT), alkaline phosphatase (AKP), total protein (TP), total bilirubin (TBil), fatty liver disease (FLD), gallbladder diseases, and cholelithiasis (all *p *< 0.001). Age‐adjusted logistic regression confirmed these associations for albumin, GGT, AKP, TP, and FLD. Although the network analysis did not identify any biomarkers with significant *p*‐values, TBil (betweenness = 8.0), TP (betweenness = 5.0), FLD (betweenness = 3.5), and AKP (betweenness = 3.5) were selected as key biomarkers based on their network centrality and established roles in hepatobiliary diseases [26, 27, 28], reflecting their relevance to CVD [8, 27, 29].

In the thyroid dimension, significant correlations with CVD were observed for urine iodine (UI), erythrocyte sedimentation rate (ESR), thyroid adenoma, and glial retention (*p *< 0.01). Network analysis identified thyroid peroxidase antibodies (TPO‐Ab) (betweenness = 10.92), hyperthyroidism (betweenness = 7.85), and thyroxine (T4) (betweenness = 17.126) as the top three indices. TPO‐Ab was excluded due to its limited availability in routine screenings. T4 and thyroid‐stimulating hormone (TSH) were prioritized as representative indicators for hyperthyroidism, as both are commonly measured during standard medical assessments [30]. Thyroid disease and related dysfunction were included due to their significant association with CVD through mechanisms such as dyslipidemia [31].

In the context of renal function, the estimated glomerular filtration rate (eGFR) was calculated using the CKD‐EPI creatinine equation 2021 [32]. Correlation analysis revealed significant associations between CVD and levels of serum uric acid (UA), serum and urine creatinine, and eGFR [33]. Although the network analysis did not identify any biomarkers with statistically significant *p*‐values, microalbuminuria, with the highest betweenness centrality (betweenness = 6.0), was identified as a central indicator. Additionally, eGFR was considered crucial due to its widespread use and significance in kidney dysfunction and blood pressure (BP) regulation [34]. Both eGFR and microalbuminuria, as essential indicators for assessing kidney function, are inextricably linked to the risk of CVD events and all‐cause mortality [35, 36, 37, 38].

Bone density, the indicator related to skeletal metabolism, was found to have significant correlations with CVD and CKD (*p *< 0.01) and was also included as one of the key indicators in downstream analysis [39, 40, 41].

**FIGURE 3:**
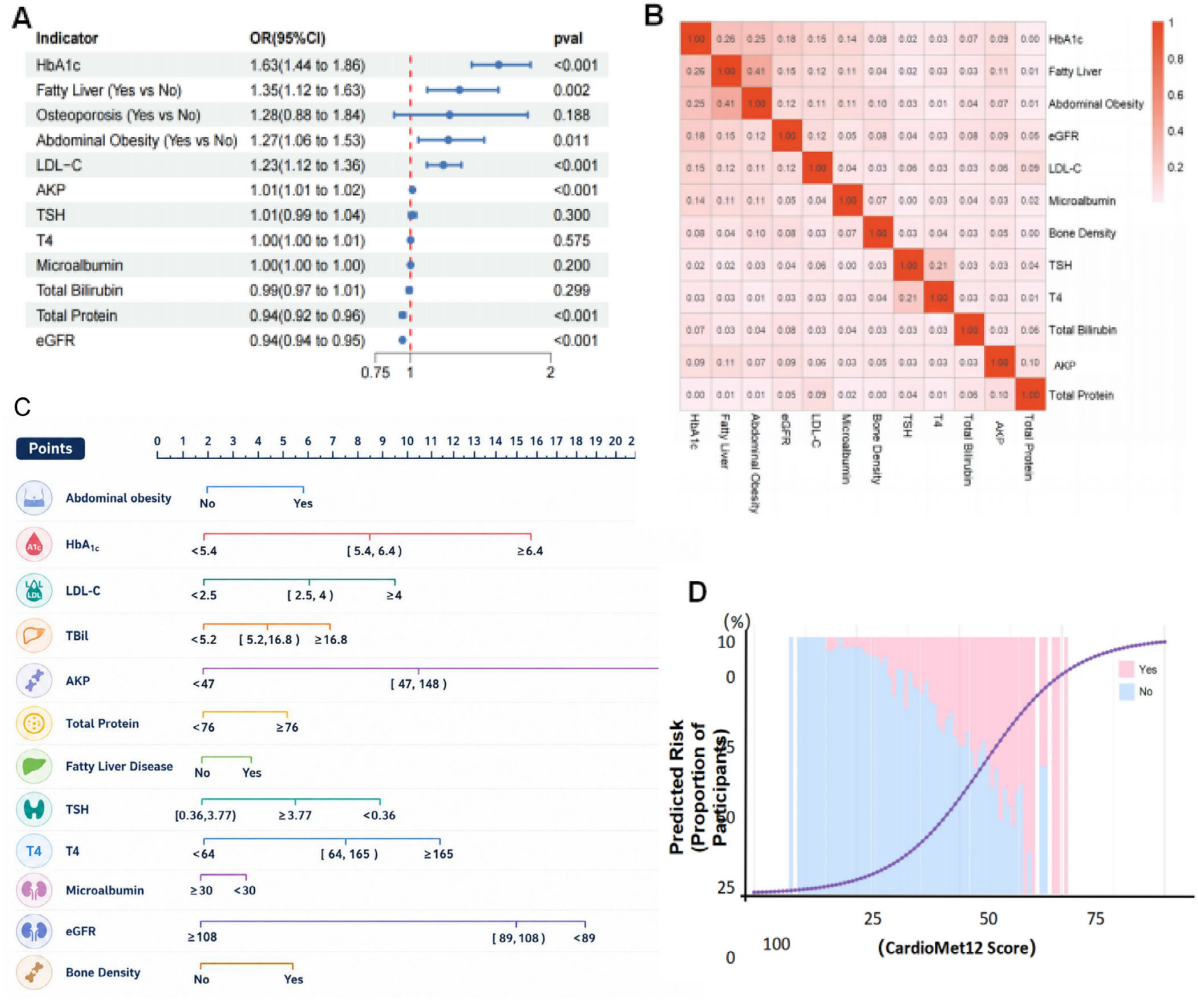
Analysis of 12 indicators involved in CardioMet12. (A) Results of logistic regression of 12 selected indicators and CVD. (B) The correlation heatmap presenting the interaction relationship of 12 selected indicators. (C) CardioMet12 CVD risk scoring system. (D) Estimated prevalence of CVD across different CardioMet12 scores. HbA_1c_, glycated hemoglobin; FLD, fatty liver disease; LDL‐C, low‐density lipoprotein cholesterol; TBil, total bilirubin; AKP, alkaline phosphatase; TSH, thyrotropin; T_4_, thyroxine; eGFR, estimated glomerular filtration rate; OR, odds ratio; CI, confidence interval.

The pairwise correlations were calculated among the 12 selected indicators, in which Cramér's V revealed low concordance overall (maximum correlation = 0.41 between FLD and abdominal obesity), thus confirming that the indicators were comprehensive and non‐collinear (Figure 3B). Logistic regression results for each indicator's association with CVD status are presented in a forest plot in Figure 3A. Betweenness centrality and *p*‐values for each indicator are detailed in Table S3.

The indicators were compared with those in 17 existing CVD prediction models and five MetS criteria (Tables S4 and S5). Traditional models such as Framingham, SCORE2, and PCE predominantly rely on biomarkers associated with dyslipidemia and glucose metabolism. Although more recently developed models like PREVENT and QRISK3 have broadened the scope to include renal function, their overall predictor sets remain largely confined to conventional cardiovascular risk factors. In contrast, CardioMet12 demonstrates substantially broader coverage and greater metabolic specificity, with no existing model achieving a comparable level of comprehensiveness. After excluding age and sex, only five CardioMet12 indicators were included in prior models, underscoring the expanded scope and specificity of the selected indicators.

In summary, integrating expert knowledge with statistical analyses allowed us to identify 12 key indicators linking the metabolic network to CVD. These indicators were used to design a scoring system for CKM screening and detection, highlighting the interconnected nature of the multidimensional metabolic health network and pan‐vascular outcomes.

### 2.4 CVD Screening Scoring System (CardioMet12) Based on 12 Indicators

The CardioMet12 was developed to screen for prevalent CVD using 12 selected central indicators (Figure 3C). Each indicator was assigned points based on clinically relevant thresholds, with higher scores indicating increased positivity of CVD diagnosis. Notably, eGFR, AKP, and HbA_1c_ were the three factors contributing most to the final scores (Figure 3C). A 10% perturbation was applied around established clinical cutoff values to optimize these thresholds, followed by the use of a genetic algorithm [42, 43] to identify the optimal thresholds. This approach was specifically designed to better accommodate population‐specific variations in the Chinese cohort.

Given the small sample size of patients with an eGFR below 60 mL/min/1.73 m^2^, indicative of CKD, and the fact that seven out of these 10 patients had CVD, this subgroup was excluded from the scoring system development to avoid potential biases. Nevertheless, the strong association between impaired kidney function and cardiovascular risk was acknowledged. While sex and age are well‐established factors correlated with CVD, they were excluded from the scoring system to focus solely on modifiable metabolic factors, which are more actionable for intervention.

The total score was calculated by summing the points for each indicator, yielding an overall assessment. Figure 3D displays the possibility and the distribution of the participants across different scores, demonstrating that as scores increase, the evaluated risk and the observed proportion of CVD patients also rise. Overall, individuals scoring ≥56 had an observed CVD prevalence exceeding 50%.

**FIGURE 4:**
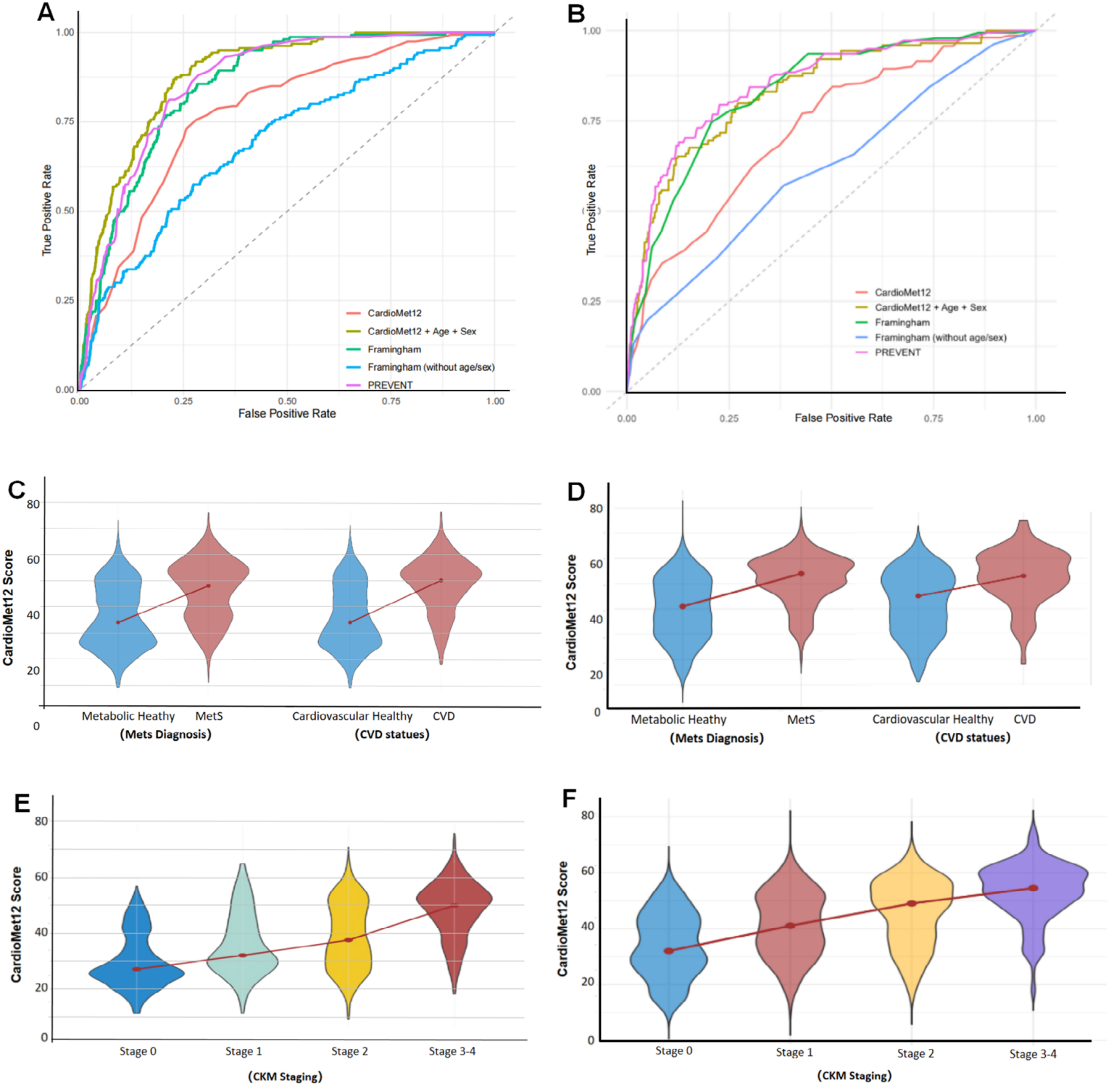
Validation in the STONE cohort and NHANES population. (A) ROC of CardioMet12 in the test set. (B) ROC of CardioMet12 in NHANES population. (C) Correlation of CardioMet12 score with MetS diagnosis and CVD statues in STONE. (D) Correlation of CardioMet12 score with MetS diagnosis and CVD statues in NHANES. (E) Comparing CardioMet12 with CKM staging in STONE. (F) Comparing CardioMet12 with CKM staging in NHANES. ROC, receiver operating characteristic curve; MetS, metabolic syndrome; CVD, cardiovascular diseases; CKM, cardiovascular‐kidney‐metabolic syndrome; NHANES, National Health and Nutrition Examination Survey.

Evaluation on the test set demonstrated acceptable positivity of CVD diagnosis, with an area under the receiver operating characteristic curve (AUC) of 0.771 (95% confidence interval [CI]: 0.733, 0.808), with a sensitivity of 0.756 (95% CI: 0.681, 0.816) and a specificity of 0.732 (95% CI: 0.696, 0.759) (Figure 4A). Bootstrap internal validation using 200 resamples estimated a mean AUC optimism of 0.013 (95% percentile interval: −0.005 to 0.030). After subtracting the mean optimism from the apparent AUC, the optimism‐corrected AUC was 0.758 (95% percentile interval, 0.741 to 0.776).The corresponding optimism‐corrected Brier score and calibration slope were 0.158 (95% percentile interval, 0.152 to 0.165) and 0.937 (95% percentile interval, 0.855 to 1.028), respectively (Table S6 **)**. Beyond discrimination, we evaluated the clinical utility and calibration of the model. Decision curve analysis (DCA) demonstrated that CardioMet12 outperformed the treat‐all strategy at the clinically relevant threshold of about 21% (corresponding to the sample CVD prevalence). Calibration plots showed good agreement between estimated probabilities and observed outcomes of the model (Table S7 and Figures S1 and S2).

The CardioMet12 offers a practical and reliable method for CVD risk stratification, using only 12 modifiable indicators that are obtained from routine examinations, allowing clinicians to identify high‐risk individuals through a comprehensive metabolic profile. This tool can support early screening and personalized management to reduce CVD risk in vulnerable populations.

### 2.5 Head‐to‐Head Benchmarking With PREVENT and Framingham

To contextualize CardioMet12 against established scoring systems, five models were evaluated on the STONE test set: CardioMet12, CardioMet12_E_ (CardioMet12 with age and sex) via a logistic model, the Framingham risk score, a Framingham variant with age and sex removed, and PREVENT. For comparability, classification cut‐points were set by Youden's index on each ROC curve. The overlaid ROC curves are shown in Figure 4A. CardioMet12 achieved an AUC of 0.771. Adding age and sex improved performance to an AUC of 0.883, with a sensitivity of 0.881, specificity of 0.764, and accuracy of 0.789. PREVENT achieved an AUC of 0.863 (0.812 sensitivity, 0.784 specificity, and 0.790 accuracy), and the Framingham general CVD score reached an AUC of 0.854 (0.856 sensitivity, 0.715 specificity, and 0.744 accuracy). Removing age and sex from Framingham degraded performance to an AUC of 0.691 (0.569 sensitivity, 0.732 specificity, and 0.698 accuracy). Taken together, CardioMet12 with age and sex provided the strongest discrimination on the test set, exceeding PREVENT by 0.02 AUC and Framingham by 0.029 AUC, while the demography‐free CardioMet12 remained competitive despite using only modifiable biomarkers.

The findings suggest a pragmatic two‐tier deployment. In clinical settings where demographics are readily available, CardioMet12_E_ can support triage and diagnostic decision‐making. For population screening and longitudinal self‐monitoring, CardioMet12 only preserves focus on modifiable biology and can be repeated to track response to lifestyle or therapy without the confounding impact of immutable factors.

### 2.6 Validation of CardioMet12 in MetS, CKM Staging, and the Nhanes Population

After evaluating the clinical utility and effectiveness of CardioMet12 in screening the diagnostic potential of CVD, CardioMet12 was then compared with established diagnostic criteria for MetS and the CKM stages. We applied established clinical criteria to our dataset after thorough data preprocessing. For MetS diagnosis, we used the National Cholesterol Education Program Adult Treatment Panel III (NCEP ATP III) guidelines [7], identifying individuals who met at least three out of five specific criteria related to abdominal obesity, TG, HDL‐C, BP, and FPG. For CKM staging, we categorized participants into Stage 0, 1, 2, and 3–4 based on parameters including BMI, abdominal obesity, glycemic status, BP, lipid profile, CKD, and CVD [8]. As our focus is on the general population, Stages 3 and 4 were combined into one group.

A positive correlation was observed between CardioMet12 scores and CKM staging. Specifically, individuals with higher CardioMet12 scores were more likely to meet MetS criteria (*p *< 0.001) and to be classified in higher CKM staging (*p *< 0.001) (Figure 4C,E and Table S8).

Data from NHANES 2007–2012 were also utilized for further validation (Table S9 and Figure S3). The CKM staging definitions adapted from the available NHANES data were applied in this study [44]. The baseline characteristics of 1620 participants are presented in Table S10, representing approximately 55 million US adults aged 20 years or older. The analytical sample (*n* = 1620) represents 9.1% of eligible participants (*n* = 17,713), as the majority were excluded due to NHANES planned subsampling of specific laboratory measures. Included participants were younger (median 45 vs. 50 years) with lower CVD prevalence (7.3% vs. 11.9%) compared to excluded participants (Table S11), suggesting that our discrimination estimates may be conservative. Among all participants, 119 were diagnosed with CVD, with a weighted mean age of 43.11, and 50.4% were male. Initial analyses showed significant differences in CardioMet12 included indicators between individuals with and without CVD, except for TSH, TP, and T4.

The weighted results demonstrated a significant difference in CardioMet12 scores between individuals with and without CVD (Figure 4D). The weighted mean total score for individuals without CVD was 42.42, whereas for those with CVD, it was 52.29 (*p *< 0.001). In addition, the relationship between CardioMet12 scores and MetS was explored. The weighted mean total score for individuals without MetS was 39.91, while for those with MetS was 51.46, with a significant *p *< 0.001 (Figure 4D). Further analysis of total scores across different CKM stages also demonstrated significant associations (Figure 4F and Table S12).

Similarly, five models were evaluated on the NHANES test set (Figure 4B), and similar findings were observed, where CardioMet12E achieved an AUC of 0.827. PREVENT provided the numerically strongest discrimination on the NHANES test set, while CardioMet12E showed discrimination that was not statistically significantly different from PREVENT (*p* = 0.132). Detailed performance metrics are presented in Table S13. Calibration analyses indicated that raw CardioMet12 probabilities required recalibration in NHANES (Brier score = 0.148; Hosmer–Lemeshow *p* < 0.001), whereas recalibration improved the Brier score to 0.0646. CardioMet12E showed the most favorable calibration metrics (Brier score = 0.0591; Hosmer–Lemeshow *p* = 0.595); DCA, calibration, and survey‐weighted AUC results are presented in Tables S14 and S15 and Figures S4 and S5.

### 2.7 Sensitivity Analysis

Results of sensitivity analyses evaluating CardioMet12 performance in the STONE and NHANES populations are detailed in Tables S16 and S17, respectively.

## 3 Discussion

This work presents CardioMet12, a 12‐item diagnostic score derived from a city‐wide, probability‐sampled cohort of residents in Shanghai, China, and externally validated in NHANES by interrogating seven interconnected metabolic axes (i.e., adiposity, glucose regulation, lipoprotein transport, hepatobiliary metabolism, thyroid activity, renal filtration, and bone turnover) through differential network analysis, the relational “gatekeepers” whose patterns most clearly distinguished individuals with CVD were identified. Consolidating these laboratory and anthropometric markers into a single metric produced a tool that is both parsimonious and mechanistically coherent.

Recently, substantial attention has been directed toward the relationship between MetS and CVD [7, 45], and diagnostic criteria have been developed to address these associations. Current CVD prediction models primarily focus on the aspects of glucose and lipid metabolism disorders. Therefore, TC, HDL‐C, HbA_1c_, FPG, and 2hPG are the most commonly used biomarkers. Age, sex, and smoking status, recognized as significant contributors to CVD risk, are included in most scales and account for a significant portion of the scoring. These scales have been commonly applied in Western populations, especially the Framingham Heart Study model and the Pooled Cohort Equations (PCE) [2, 46]. Such systems rely heavily on fixed demographics and a narrow set of lipid or glycemic values [2, 3, 46]. These models discriminate well between people, but respond poorly to change within a person; the risk contribution of age, in particular, can overshadow the improvements achieved through lifestyle or pharmacotherapy. Indeed, most commonly used systems place excessive emphasis on demographic factors, such as sex, age, and social determinants of health (Table S4), which are generally non‐modifiable. CardioMet12 addresses this limitation by excluding non‐modifiable demographics and focusing exclusively on indices that can change within weeks to months. Consequently, a decline in the score signals genuine biological improvement rather than the artefact of a patient simply “getting older.” The broader biochemical palette also captures cross‐talk between endocrine, hepatic, and renal pathways that converge on vascular injury, an integration absent from previous risk charts. Currently published models are not comprehensive enough in addressing all metabolic dysfunctions. While the latest PREVENT model incorporates kidney function indicators [47], other critical aspects of metabolic health, such as thyroid function and bone metabolism, are still overlooked. Importantly, every component of CardioMet12 is measured in routine practice, while the reliance on uncommon biomarkers restricts the accessibility of these scoring systems, for instance, high‐sensitivity C‐reactive protein (hsCRP) in the Reynolds Risk Score [48] and Apolipoprotein B/Apolipoprotein A‐I (ApoB/AI) in the INTERHEART Modifiable Risk Score [48, 49, 50, 51], creating additional costs and barriers to the widespread use of CVD screening.

Based on epidemiological data from China, the present study classified routine biomarkers into seven metabolic health aspects and established a metabolic network, analyzed and used to identify key indicators in each metabolic dysfunction aspect. Metrics such as LDL‐C, HbA_1c_, and BMI were identified as core metrics for CVD risk assessment in the present network analysis, consistent with the conclusions of many epidemiological studies [52, 53, 54]. These indicators informed the development of the CardioMet12, which addresses the limitations of current CVD evaluation approaches in important ways. The main innovation of the present study was the use of a network analysis to identify 12 biomarkers that encompassed seven metabolic function categories, thus more broadly examining the factors involved in CVD pathogenesis. By spanning seven metabolic categories, the selected biomarkers provide a broader perspective on CVD risk factors, encompassing not only classical lipids and glucose metabolism but also protein turnover, renal function, hepatic status, thyroid hormones, and bone health. This multidimensional approach aligns with recent advances in network medicine that highlight the interconnected nature of biological systems in cardiovascular disease [55, 56].

CardioMet12 was designed as a screening tool for prevalent CVD rather than a prospective risk prediction model. Notably, comparisons with Framingham and PREVENT were performed primarily to benchmark relative discrimination for the same binary outcome (prevalent CVD), rather than to evaluate these models in their intended 10‐year absolute risk prediction settings. The AUC comparison therefore reflects discriminative ability, not equivalent clinical deployment scenarios. Rather than competing with established prospective risk prediction engines, CardioMet12 complements the existing tools by focusing on the working‐age population (18–65 years) where metabolic risk factors are most modifiable and early intervention yields the greatest long‐term benefit. By targeting this demographic, CardioMet12 fills a specific gap between primordial prevention in youth and event‐driven risk assessment in older adults, aligning with contemporary emphasis on life course cardiovascular health. Future longitudinal follow‐up will evaluate its predictive utility.

Despite these strengths, several limitations should be acknowledged. First, we recognized that our external validation spanned distinct CVD phenotypes, as the definition of CVD in STONE and NHANES was not fully equivalent. This phenotypic heterogeneity may attenuate or alter discrimination and calibration to some extent. However, rather than compromising validity, this divergence offers valuable insight into model transportability across different disease constructs, from symptomatic clinical endpoints to preclinical imaging markers. Second, external validation involved inherent demographic and methodological contrasts between the STONE and NHANES populations. Four of 12 CardioMet12 components required methodological adaptation, specifically, fatty liver index, spot urine microalbumin, ATP III waist cutpoints, and dual‐energy x‐ray absorptiometry (DXA) bone density, which potentially introduces measurement heterogeneity. Finally, although differential network analysis clarifies how the seven metabolic systems intercorrelate, it offers limited insight into the molecular pathways that generate these links.

Hence, CardioMet12 leverages differential network analysis to distil seven metabolic systems into 12 routinely measured “gatekeeper” biomarkers, producing a dynamic, laboratory‐based gauge of cardiometabolic risk that omits immutable demographics and relies only on widely available assays. A freely accessible online calculator has been implemented (http://cz‐endo.com) (detailed in File S1).

## 4 Conclusion

Using a novel epidemiological cohort and metabolic network analysis method, metabolic health factors were classified into seven facets, and 12 routine indicators were identified to represent MetS and CVD risks and to build the CardioMet12 system using the STONE and NHANES populations. CardioMet12 aims to support early detection and targeted intervention for CVD risk. This study used network analysis to identify complex associations between metabolism and CVD. Hence, CardioMet12 has the potential to be a comprehensive and effective tool for assessing CVD metabolic burden, aiding in early screening and targeted intervention.

## 5 Methods

### 5.1 Participants and Sampling Strategy

This retrospective, population‐based study was conducted using the data from the STONE study, which was performed among the residents of Shanghai, encompassing 18 districts and 242 community health service centers. The study was approved by the Shanghai Changzheng Hospital Ethics Committee (2016SL039). In the initial study, a three‐stage stratified sampling strategy was employed to secure a sample representative of Shanghai's demography from December 2016 to November 2018. Considering the demographic distribution across Shanghai, 10 community health centers were randomly selected, with four located in urban areas, three in urban–rural areas, and three in rural areas. Then, the residential communities provided information on the composition of each household's population, and a random sampling approach was applied to select the households. Ultimately, the KISH grid method was employed to determine the individuals to be surveyed [57].

Considering sex as a biological variable, males and females were randomly involved in the epidemiological survey. The inclusion criteria were (i) individuals without severe diseases; (ii) living in the selected community for more than 6 months; and (iii) aged between 18 and 65 years. The exclusion criteria were (I) pregnancy or abortion less than 6 months before; (II) severe liver or kidney failure‐related diseases; (III) other severe autoimmune diseases, solid or hematologic malignancy, or other high‐risk conditions for mortality; or (IV) declined participation. Ultimately, 4066 individuals were included in the present analysis (Figure 1).

All procedures performed in the study were in accordance with the ethical standards of the institutional research committee and with the 1964 Helsinki Declaration and its later amendments or comparable ethical standards. Informed consent was obtained from all individuals included in the study. The study was approved by the Shanghai Changzheng Hospital Biomedical Research Ethics Committee (approval #2016SL039).

### 5.2 Data Collection and Measurements

In every community health center, comprehensive surveys, anthropometric measurements, blood samples, urine samples, sonography, and dual‐energy x‐ray absorptiometry (DXA) were collected. Trained healthcare providers conducted confidential interviews with participants to fill out a questionnaire, which included information on sex, age, educational attainment, marital status, household composition, employment status, birthplace, number of children, nutritional supplement use, weight control efforts, life rhythm disruptions, contraceptive use history, alcohol consumption history, smoking habits, and mental health status.

### 5.3 Anthropometric Measurement

All participants underwent measurements for height, weight, WC, and blood pressure by the trained staff. Waist circumference was measured at the horizontal level of the navel. Blood pressure was measured after a 5‐min rest.

### 5.4 Blood Samples

Blood samples were collected in the morning following an overnight fast of at least 8 h. Biochemical analyses included markers associated with dyslipidemia, glucose metabolism disorders, CKD, liver function, and thyroid function (Table S18). In terms of lipid metabolism, TC, TG, HDL‐C, and LDL‐C were analyzed. As for glucose metabolism, eight biochemical markers, including FPG, fasting insulin (FINS), fasting C‐peptide, 2hPG, 2‐h insulin, 2‐h C‐peptide, HbA_1c_, and HOMA‐IR, were analyzed. Serum creatinine was also measured for the evaluation of kidney function. In the category of hepatobiliary diseases, the biochemical assays provided data on liver function markers, including total and direct bilirubin levels, Alb, aspartate aminotransferase (AST), alanine aminotransferase (ALT), GGT, AKP, and TP levels. A variety of markers related to thyroid function were conducted, encompassing levels of triiodothyronine (T3), T4, free triiodothyronine (fT3), free tetraiodothyronine (fT4), thyroxine‐binding globulin (TBG), TSH, thyrotropin receptor antibody (TR‐Ab), thyroglobulin (Tg), thyroglobulin antibody (Tg‐Ab), TPO‐Ab, and immune markers such as Complement Component 3, Complement Component 4, and ESR.

### 5.5 Urine Analysis

Midstream urine samples were collected and tested for UI, UA, urine creatinine, microalbumin, and albumin‐to‐creatinine ratio (ACR).

### 5.6 Auxiliary Examinations

Thyroid ultrasound examinations were conducted using probes of 5–10 MHz. Carotid ultrasound examinations were performed using three different high‐resolution ultrasound systems: (i) GE Voluson E8 (GE Healthcare, USA) with a 3–8 MHz linear array transducer; (ii) Esaote MyLab 90 (Esaote, USA) with a 3–9 MHz linear array transducer; and (iii) Hitachi‐Aloka Prosound 2 portable unit (Hitachi‐Aloka Medical, Japan) with a 5–13 MHz linear array transducer. Abdominal ultrasound was performed by experienced sonographers utilizing a 3.5 MHz probe on the liver and gallbladder of the participants. It involved scrutinizing the liver for signs of fatty infiltration and examining the gallbladder for polyps and stones, as well as the gallbladder wall and bile ducts. In addition, a detailed assessment of the structure and function of the thyroid was conducted, searching for evidence of conditions like nodular goiter, Hashimoto's thyroiditis, thyroid adenoma, thyrotoxicosis, thyroid nodules with suspected malignancy, colloid retention, and the postoperative scenario following thyroidectomy. All ultrasound images were interpreted by two experienced sonographers with attending physician‐level qualification or above (more than 5 years of advanced ultrasound experience). To ensure diagnostic accuracy and minimize inter‐observer variability, all readings were subsequently reviewed and verified by a senior physician with associate chief physician rank or higher. Measurements of bone mineral density (BMD) were performed using DXA at the conclusion of the study.

### 5.7 Data From NHANES

The NHANES is a nationally representative survey conducted in the United States, aimed at evaluating the health and nutritional status of the general population. For the validation of the model developed here, the NHANES data collected between 2007 and 2012 were utilized.

Twelve relevant variables were selected from the CardioMet12 for this analysis according to their NHANES variable codes (Table S9). The eGFR was estimated using the 2021 CKD‐EPI creatinine equation. FLD was determined using the Fatty Liver Index (FLI), where an FLI score ≥60 was indicative of FLD, and scores less than 60 represented the absence of FLD [58]. Osteoporosis was defined as BMD less than −2.5 SD below the young adult reference population using dual‐energy x‐ray absorptiometry and WHO criteria [59]. CVD history was based on self‐reported diagnoses of heart failure, coronary heart disease, angina, myocardial infarction, or stroke [60].

### 5.8 Clinical Definitions and Diagnostic Criteria

Cardiovascular diseases in this study included the presence of either clinical or subclinical CVD. Clinical CVD was identified based on self‐reported history of cardiovascular disease or current use of CVD‐related medications through a questionnaire survey. Subclinical CVD was defined as ultrasound‐defined carotid atherosclerosis, manifested as presence of artery stenosis, wall plaque, atherosclerosis, or related conditions detected via carotid ultrasound. Obesity was defined as a BMI of ≥28 kg/m^2^, along with a waist circumference of ≥85 cm in women/90 cm in men [61]. If any of the following fasting venous plasma test criteria were met, dyslipidemia was diagnosed: TC ≥6.2 mmol/L, TG ≥2.3 mmol/L, LDL‐C ≥4.1 mmol/L, and HDL‐C less than 1.0 mmol/L [62]. Diabetes was defined as the presence of typical diabetes symptoms (such as excessive thirst and urination, increased appetite, and unexplained weight loss) along with random blood glucose ≥11.1 mmol/L, or FBG ≥7.0 mmol/L, or OGTT 2‐h blood glucose ≥11.1 mmol/L following a glucose load, or HbA_1c_ ≥6.5% [63]. HOMA‐IR index was used to define insulin resistance and is calculated with the following formula: HOMA‐IR = FBG (mmol/L) × Ins (mU/L)/22.5 [64]. Wall plaque, detected by carotid ultrasound, was defined as localized structural changes that protrude into the arterial lumen and exceed the surrounding intima‐media thickness (IMT) by at least 0.5 mm or 50%, or IMT greater than 1.5 mm [65]. CKM syndrome was classified into Stage 0 to Stage 4, including no risk factors, excess/dysfunctional adipose tissue, metabolic risk factors, CKD, subclinical CVD in CKM syndrome, and clinical CVD in CKM syndrome [45]. CKD was stratified into Stages G1 through G5, with the stages reflecting a descending scale of glomerular filtration rate (GFR) values [66].

### 5.9 Detection of Central Indicators Within Each Metabolic Domain

A differential network analysis was performed to compare the correlation structure of each domain between participants with and without CVD to isolate the biomarkers that orchestrate the metabolic perturbations accompanying CVD. The candidate pool was first restricted to variables exhibiting less than 30% missingness and adequate dispersion (≤95 % of observations in any single category), thereby ensuring stable correlation estimates. Within each metabolic domain and CVD stratum, pairwise associations were computed using Pearson correlation, chi‐square tests, and one‐way ANOVA. To evaluate the impact of multiple testing, two edge‐thresholding regimens were applied in parallel: (i) Benjamini–Hochberg FDR‐corrected *q* < 0.05 (primary analysis), and (ii) unadjusted *p *< 0.05 (sensitivity analysis). The resulting *p*‐values populated two symmetric association matrices, *p_case_* and *p_control_*, for every metabolic domain. Each matrix was thresholded at *α* = 0.05 to generate the binary adjacency matrices *A*_case_ and *A*_control_ that encoded edges representing statistically significant relationships. As the analysis involved both continuous–continuous pairs and numerous categorical variables, a domain‐specific “change network” was then derived as the element‐wise exclusive‐OR (⊕) of these two adjacency matrices, *AΔ = A*_case_ ⊕ *A*_control_, so that an edge was present only if its significance status differed between cases and controls. Notably, this approach identifies shifts in statistical significance status rather than direct changes in correlation magnitude. For each domain network, we report edge count, edge density (observed edges/possible edges), and network sparsity (1 − Density) (Table 2). For every node in *A_Δ_*, betweenness centrality was calculated, a measure of a variable's capacity to broker information flow across the network by lying on the shortest paths between other pairs of nodes. The statistical significance of each betweenness estimate was assessed through a 500‐fold permutation procedure that reran the entire pipeline after randomly shuffling CVD status labels. This yielded an empirical null distribution of betweenness values from which two‐tailed permutation *p*‐values and 95% confidence intervals were obtained. Indicator selection within each domain followed four pre‐specified, sequentially applied criteria: (i) statistical relevance with CVD: permutation‐adjusted *p*‐value was less than 0.05; (ii) network centrality: the variable with the highest betweenness in the differential change network was preferred; (iii) clinical relevance: the candidate must have an established mechanistic or epidemiological link to CVD; and (iv) routine availability: the biomarker must be obtainable in standard health screenings without specialized assays. When the network‐top variable was not clinically interpretable or not routinely available, it was replaced by the highest‐ranked alternative that satisfied the statistical relevance, clinical relevance, and routine‐availability criteria. The rationale for each domain is detailed in Table S2, and swap‐in sensitivity analyses replacing overridden indicators with their network‐top alternatives are reported in Table S19.

### 5.10 CardioMet12 Design

Missing data for the 12 metabolic biomarkers were imputed using the multivariate imputation by chained equations (MICE) method with predictive mean matching, implemented through the mice R package (version 3.16.0). Multiple imputed datasets were generated and combined to minimize bias and enhance the robustness of the statistical analyses.

The scoring system was developed through a structured process involving data preparation, score generation, and model validation. The dataset was divided into training, validation, and test sets, representing 70%, 10%, and 20% of the data, respectively. The training and validation sets were optimized using up‐and‐down sampling techniques to address class imbalances and improve model performance. The pre‐selected variables were used to generate initial scores, with each category's score obtained using the AutoScore R package (version 1.0.0) through logistic regression for score weighting [67]. We first applied quantile‐based cutoff points to each variable. A 10% perturbation was introduced around established clinical cutoff values to optimize these thresholds. It allowed for a refined adjustment of thresholds for key biomarkers, guided by the area under the curve (AUC) of the receiver operating characteristic (ROC) to identify optimal values within the validation set with the application of a genetic algorithm implemented using the GA R package (version 3.2.4) [43]. This approach, specifically tailored to the characteristics of the Chinese cohort, resulted in more precise cutoff points that better captured population‐specific variations and improved model performance.

The final scoring model was evaluated using the test dataset, with discriminative performance assessed through ROC analysis. The total scores were mapped to cardiovascular risk probabilities, allowing for a clear interpretation of an individual's risk based on their calculated score.

## Author Contributions

All authors read and approved the manuscript. T.L., W.T., J.J.Z., J.Z., and Y.Q.S. designed the study. T.L., Q.L., Y.S., X.Z., and H.H. contributed to experimental design and sample size calculation. T.L., W.T., Q.C., and J.Z. took part in the acquisition of data. T.L., X.C., J.F.Z., Q.C., and J.Z. performed data analysis. T.L., X.C., J.F.Z., and J.Z. wrote the original draft of the manuscript and drew the figures. Y.Q.S., J.Z., and J.J.Z. reviewed and edited the final version of the manuscript. All authors have read and approved the final manuscript.

## Funding

The funding for this study was supported by the Shanghai Municipal Health Commission Grant (No.: GWIV‐27.7 to Y.Q.S.); Chongqing Municipal Science and Technology Bureau (No. CSTB2024YCJH‐KYXM0098 to J.Z.); Venture and Innovation Support Program for Chongqing Overseas Returnees (No. 203011220240002 to J.Z.); General Project of National Natural Science Foundation of China (No.: 82170858, 82470935 to T.L.); and “Dawn” Program of Shanghai Education Commission, China (No.: 23SG33, to T.L.).

## Ethics Statement

The STONE study (Shanghai Thyroid autOimmuNity: an Epidemiological research) was approved by the Shanghai Changzheng Hospital Biomedical Research Ethics Committee (approval #2016SL039, NCT07043166). Informed consent was obtained from all participants in the study.

## Conflicts of Interest

The authors declare no conflicts of interest.

## Supporting information




**Supporting File**: mco270967‐sup‐0001‐SuppMat.docx

## Data Availability

NHANES data are publicly available through the National Center for Health Statistics at https://www.cdc.gov/nchs/nhanes/. Consistent with our institutional ethical approvals and data sharing agreements, the individual‐level participant data of STONE population cannot be made publicly available due to confidentiality requirements and privacy protection regulations.
