# Supplementary material for: Unraveling the Metabolic‐Cardiovascular Disease Nexus: An Epidemiological Network Analysis Identifies Central Indicators
Source: MedComm (2020). 2026 Sep 3;7(9):e70967. doi: 10.1002/mco2.70967 (PMC13542700; doi:10.1002/mco2.70967)
Supplement: Supplementary file 1 [file MCO2-7-e70967-s001.docx]

**Supplementary**

Unraveling the Metabolic-Cardiovascular Disease Nexus: An Epidemiological Network Analysis Identifies Central Indicators

## Running title: CardioMet12: Metabolic-Cardiovascular Nexus

Xin-yi Cai, B.D. ^1#^, Jia-feng Zhang, M.S. ^2#^, Hao Huang, B.D. ^1^, Wei Tang, M.D. ^1^, Qian Lv, M.S. ^1^, Yan Song, M.S. ^1^, Xing-xing Zhang, M.S. ^1^, Quan-cai Cai, M.D. ^3^, Jian Zou, Ph.D. ^4*^, Yong-quan Shi, M.D.^1*^, Tuo Li, M.D. ^1*^

^1^ Department of Endocrinology, Changzheng Hospital, Shanghai 200003, China

^2^ Department of Endocrinology, No. 905 Hospital of PLA Navy, Shanghai Changzheng Hospital, Shanghai 200052, China.

^3^ Department of Epidemiology, Changhai Hospital, Shanghai 200433, China

^4^ Department of Statistics, School of Public Health, Chongqing Medical University, Chongqing 400016, China

^#^ These authors contributed to the work equally.

^*^ Correspondence to :

Tuo-Li, M.D., Department of Endocrinology, Changzheng Hospital, Shanghai 200003, China; Tel (O): +86-021-81886821; E-mail: drlituo@smmu.edu.cn (T.L.)

Yong-quan Shi, M.D., Department of Endocrinology, Changzheng Hospital, Shanghai 200003, China; Tel (O): +86-021-81886821; E-mail: [young.stone@163.com](mailto:young.stone@163.com) (Y.S.)

Jian Zou, Ph.D., Department of Statistics, School of Public Health, Chongqing Medical University, Chongqing 400016, China; E-mail: [jianzou@cqmu.edu.cn](mailto:jianzou@cqmu.edu.cn) (J.Z.)

The authors have declared that no conflict of interest exists.

**Supplementary File**

###### Online implementation of CardioMet12

The open-access calculator (http://cz-endo.com/) is delivered as a modular, bilingual web application. All user inputs (12 routinely measured biomarkers) are range-checked in the browser, transmitted via HTTPS, and re-validated server-side. To handle incomplete submissions, the back-end applies a K-Nearest-Neighbor (KNN) routine: if three or fewer variables are missing, z-standardized Euclidean distances are calculated against the reference STONE cohort, and the 10 closest complete records (k=10) supplies substitute values. When more than three variables are missing, processing stops and the user is prompted to supply additional data. In a held-out subset of 771 STONE participants, we artificially masked 1-3 variables per individual and applied KNN imputation. The resulting scores demonstrated near-identical concordance with those derived from complete data (ICC = 0.912, 95%CI: 0.900-0.923). The completed feature vector is then passed to the published CardioMet12 coefficients, and the system returns the total score, color-coded risk category, and context-specific management advice. No personal identifiers are stored, and all code is version-controlled with automated unit tests before deployment. We commit to maintaining the CardioMet12 online calculator, and regular security updates and compatibility checks will be performed to ensure uninterrupted service.

**Supplementary Figure S1.** Calibration plots for CardioMet12 and CardioMet12_E_ in the test set of STONE population (n=7,71).

**Supplementary Figure S2.** Decision curve analysis for CardioMet12 and CardioMet12_E_ in the test set of STONE population (n=7,71).

**Supplementary Figure S3**. Flow chart illustrating the participant selection process, NHANES 2007-2012.

**Supplementary Figure S4.** Calibration plots for CardioMet12 and CardioMet12_E_ in the NHANES validation cohort (n=1,620).

**Supplementary Figure S5.** Decision curve analysis for CardioMet12 and CardioMet12_E_ in the NHANES validation cohort (n=1,620).

**Supplementary Table S1.** Baseline characteristics stratified by the development of cardiovascular diseases.

**Supplementary Table S2.** An overview of various metabolic diseases, the indices involved in each, and the selected key indicators identified through our network-based approach.

**Supplementary Table S3.** Betweenness and p-values for the variables in the CardioMet12.

**Supplementary Table S4**. Variables in current models for the prediction of cardiovascular diseases (CVD).

**Supplementary Table S5**. Variables in diagnostic criteria for MetS.

**Supplementary Table S6.** Bootstrap internal validation and optimism corrected performance of CardioMet12.

**Supplementary Table S7.** Calibration metrics and operating thresholds for CardioMet12 models in STONE population (n = 7,11).

**Supplementary Table S8**. Influence of **cardiovascular-kidney-metabolic** (CKM) staging on total points.

**Supplementary Table S9**. Assessment of 12 network indicators in CardioMet12, NHANES 2007-2012.

**Supplementary Table S10**. Baseline characteristics of U.S. adults (n = 1620) from NHANES 2007-2012, including profiles of 12 related biomarkers in CardioMet12, stratified by the presence or absence of CVD.

**Supplementary Table S11.** Comparison of baseline characteristics between included and excluded participants in the NHANES 2007-2012 cohort.

**Supplementary Table S12**. Influence of cardiovascular-kidney-metabolic (CKM) staging on CardioMet12 total points in the NHANES validation cohort (n = 1,620).

**Supplementary Table S13**. Comparative Performance of CardioMet12, Framingham, and PREVENT in NHANES population.

**Supplementary Table S14.** Calibration metrics and operating thresholds for CardioMet12 models in the NHANES validation cohort (n = 1,620).

**Supplementary Table S15.** Comparison of survey-weighted and unweighted AUC for CVD discrimination models in the NHANES validation cohort (n = 1,620).

**Supplementary Table S16.** Subgroup analysis of CardioMet12 discrimination performance by sex and age in the STONE population (n = 771).

**Supplementary Table S17.** Subgroup analysis of CardioMet12 discrimination performance by sex, age, and race/ethnicity in the NHANES validation cohort (n = 1,620).

**Supplementary Table S18**. Reagents and reference intervals for indicators.

**Supplementary Table S19**. Sensitivity analyses replacing overridden indicators with each network-top alternative.

**Supplementary Figure S1.** Calibration plots for CardioMet12 and CardioMet12_E_ in the test set of STONE population (n=7,71).

**Supplementary Figure S2.** Decision curve analysis for CardioMet12 and CardioMet12_E_ in the test set of STONE population (n=7,71). CardioMet12 outperformed the treat-all strategy at the clinically relevant threshold of about 21% (corresponding to the sample CVD prevalence).

**Supplementary Figure S3**. Flow chart illustrating the participant selection process, NHANES 2007-2012.

**Supplementary Figure S4.** Calibration plots for CardioMet12 and CardioMet12_E_ in the NHANES validation cohort (n=1,620). Predicted probability of prevalent CVD versus observed CVD prevalence across deciles of predicted risk. The diagonal dashed line represents perfect calibration. (A) CardioMet12 after intercept recalibration (calibration slope = 0.82, Hosmer-Lemeshow P = 0.049, Brier score = 0.065). Predicted probabilities were derived from STONE-derived logistic coefficients with intercept updated for baseline prevalence differences between cohorts. (B) CardioMet12+Age+Sex (Hosmer-Lemeshow P=0.595, Brier score=0.0591).

**Supplementary Figure S5.** Decision curve analysis for CardioMet12 and CardioMet12_E_ in the NHANES validation cohort (n=1,620). Net benefit is plotted across threshold probabilities from 1% to 50%, compared with the default strategies of Treat All and Treat None. CardioMet12_E_ demonstrated positive net benefit across threshold probabilities of 2%–35%.

**Supplementary Table S1.** Baseline characteristics stratified by the development of cardiovascular diseases. Cardiovascular information was obtained from ultrasound tests based on criteria including any artery stenosis, wall plaque, plaque formation, atherosclerosis, or other findings from carotid ultrasound.

| **Characteristic** | **CVD (n=922^A^)** | **without CVD (n = 3,144^A)^** | **p-value^B^** |
| --- | --- | --- | --- |
| **Sex** |  |  | <0.001 |
| Female | 454 (49%) | 1837 (58%) |  |
| Male | 468 (51%) | 1307 (42%) |  |
| **Age (years)** | 54 ± 8 | 38 ± 11 | <0.001 |
| (Missing) | 5 | 31 |  |
| **Educational Attainment** |  |  | <0.001 |
| No Formal education | 9 (1.0%) | 8 (0.3%) |  |
| Primary education | 73 (7.9%) | 81 (2.6%) |  |
| Secondary education | 637 (69%) | 1084 (34%) |  |
| Higher education | 203 (22%) | 1970 (63%) |  |
| (Missing) | 0 | 1 |  |
| **Marital Status** |  |  | <0.001 |
| Unmarried | 12 (1.3%) | 543 (17%) |  |
| Married | 864 (94%) | 2,480 (79%) |  |
| Divorced | 26 (2.8%) | 91 (2.9%) |  |
| Widowed | 20 (2.2%) | 23 (0.7%) |  |
| Other | 0 | 5 (0.2%) |  |
| (Missing) | 0 | 2 |  |
| **Household composition** |  |  | <0.001 |
| Living alone | 44 (4.8%) | 147 (4.7%) |  |
| Core family | 856 (93%) | 2,834 (90%) |  |
| Extended family | 17 (1.8%) | 87 (2.8%) |  |
| Other | 3 (0.3%) | 71 (2.3%) |  |
| (Missing) | 2 | 5 |  |
| **Employment status** |  |  | <0.001 |
| Student | 0 | 84 (2.7%) |  |
| Employed | 465 (50%) | 2,565 (82%) |  |
| Unemployed/on vacation | 39 (4.2%) | 132 (4.2%) |  |
| Homemaker | 39 (4.2%) | 84 (2.7%) |  |
| Retired | 379 (41%) | 278 (8.8%) |  |
| (Missing) | 0 | 1 |  |
| **Place of birth** |  |  | <0.001 |
| Shanghai | 770 (84%) | 2,330 (74%) |  |
| Other | 150 (16%) | 812 (26%) |  |
| (Missing) | 2 | 2 |  |
| **Household size** | 4 ± 1 | 4 ± 1 | 0.2 |
| (Missing) | 1 | 7 |  |
| **Maternal age at birth** | 26.4 ± 5.3 | 26.6 ± 4.6 | 0.4 |
| (Missing) | 263 | 306 |  |
| **Number of children** |  |  | <0.001 |
| Not married | 29 (3.1%) | 757 (24%) |  |
| Pregnant | 1 (0.1%) | 10 (0.3%) |  |
| One child | 774 (84%) | 2109 (67%) |  |
| Two or more children | 118 (13%) | 268 (8.5%) |  |
| **Nutritional supplement use** | 283 (31%) | 888 (28%) | 0.14 |
| (Missing) | 1 | 1 |  |
| **Weight control efforts** | 132 (14%) | 693 (22%) | <0.001 |
| (Missing) | 3 | 1 |  |
| **Life rhythm disruptions** |  |  | <0.001 |
| Never | 765 (83%) | 1996 (64%) |  |
| Rarely | 94 (10%) | 705 (23%) |  |
| Occasionally | 42 (4.6%) | 272 (8.7%) |  |
| Frequently | 17 (1.9%) | 143 (4.6%) |  |
| (Missing) | 4 | 28 |  |
| **Contraceptive use history** | 325 (41%) | 939 (34%) | <0.001 |
| (Missing) | 129 | 373 |  |
| **Smoking history** |  |  | <0.001 |
| Non-Smoker | 609 (66%) | 2515 (80%) |  |
| Former Smoker | 74 (8.0%) | 96 (3.1%) |  |
| Current Smoker | 239 (26%) | 533 (17%) |  |
| **Alcohol consumption history** | 247 (28%) | 642 (21%) | <0.001 |
| (Missing) | 26 | 51 |  |
| **Sleep-related mental health status** |  |  | 0.2 |
| No symptoms | 607 (67%) | 2030 (66%) |  |
| Mild | 202 (22%) | 741 (24%) |  |
| Moderate | 87 (9.6%) | 287 (9.3%) |  |
| Severe | 9 (1.0%) | 26 (0.8%) |  |
| Very severe | 5 (0.5%) | 5 (0.2%) |  |
| (Missing) | 12 | 55 |  |
| **Cognitive-related mental health status** |  |  | 0.8 |
| No symptoms | 660 (73%) | 2235 (72%) |  |
| Mild | 203 (22%) | 677 (22%) |  |
| Moderate | 44 (4.8%) | 153 (5.0%) |  |
| Severe | 3 (0.3%) | 19 (0.6%) |  |
| Very severe | 0 | 4 (0.1%) |  |
| (Missing) | 12 | 56 |  |
| **Emotion-Related Mental Health Status** |  |  | 0.078 |
| No symptoms | 652 (72%) | 2142 (69%) |  |
| Mild | 209 (23%) | 696 (23%) |  |
| Moderate | 37 (4.1%) | 194 (6.3%) |  |
| Severe | 10 (1.1%) | 50 (1.6%) |  |
| Very severe | 2 (0.2%) | 6 (0.2%) |  |
| (Missing) | 12 | 56 |  |
| **Somatic-Related Mental Health Status** |  |  | 0.5 |
| No symptoms | 596 (65%) | 2007 (65%) |  |
| Mild | 246 (27%) | 838 (27%) |  |
| Moderate | 65 (7.1%) | 212 (6.9%) |  |
| Severe | 3 (0.3%) | 26 (0.8%) |  |
| Very severe | 0 | 5 (0.2%) |  |
| (Missing) | 12 | 56 |  |
| ^A^ n (%); Mean ± SD  ^B^ Pearson's Chi-squared test; Wilcoxon rank sum test; Fisher's exact test | | | |

**Supplementary Table S2.** An overview of various metabolic diseases, the indices involved in each, and the selected key indicators identified through our network-based approach.

| **Diseases** | **Indices** | **Key Indicators** |
| --- | --- | --- |
| Obese | BMI, WC, Abdominal Obesity | Abdominal Obesity [1, 2] |
| Dyslipidemia | Total Cholesterol, Triglycerides, HDL-C, LDL-C, non-HDL-C | LDL-C[3, 4] |
| Glucose Metabolism | FPG, 2hPG, FINS, 2hINS, FCP, 2hCP, HbA_1c_, HOMA-IR, Diabetes Disease History, Hyperglycemia | HbA_1c_ [5, 6] |
| Hepatobiliary Diseases | FLD, TP, TBil, Direct bilirubin, Alb, AST, ALT, rGT, AKP, Gallbladder diseases (Gallbladder stone, Gallbladder crystal, Gallbladder possession, Gallbladder wall, Gallbladder duct, Gallbladder polyp, Gallbladder none), Hepatic duct, Bile duct | TBil [7], TP [8], FLD [9], AKP [10] |
| Thyroid Disorders | TSH, T3, T4, fT3, fT4, Tg-Ab, TPO-Ab, TSHR-Ab, Tg, UI, ESR, Cystic Degeneration, Nodules, Nodules CA, Adenomas, Glial Retention, Hashimoto's Disease, Hyperthyroidism | T4 [11], TSH [12] |
| Chronic Kidney Disease | UA, SCr, UCr, Microalbumin, ACR, eGFR | Microalbumin [13-15],  eGFR [16, 17] |
| Bone Metabolism | Bone Density | Bone Density [18-20] |
| Waist Circumference (WC), High-Density Lipoprotein Cholesterol (HDL-C), Low-Density Lipoprotein Cholesterol (LDL-C), Non-HDL Cholesterol (non-HDL-C), Fasting Plasma Glucose (FPG), 120-minute Postprandial Glucose (2hPG), Fasting Insulin (FINS), 120-minute Postprandial Insulin (2hINS), Fasting C-Peptide (FCP), 120-minute Postprandial C-Peptide (2hCP), Glycated Hemoglobin (HbA_1c_), Homeostatic Model Assessment of Insulin Resistance (HOMA-IR), Fatty Liver Disease (FLD), Total Protein (TP), Total Bilirubin (TBil), Albumin (Alb), Aspartate Aminotransferase (AST), Alanine Aminotransferase (ALT), Gamma-Glutamyl Transpeptidase (GGT), Alkaline Phosphatase (AKP), Thyrotropin (TSH), Triiodothyronine (T3), Thyroxine (T4), Free Triiodothyronine (fT3), Free Tetraiodothyronine (fT4), Thyroglobulin Antibody (Tg-Ab), Antithyroperoxidase Antibody (TPO-Ab), Thyrotropin Receptor Antibody (TSHR-Ab), Thyroglobulin (Tg), Urinary Iodine (UI), Erythrocyte Sedimentation Rate (ESR), Uric Acid (UA), Serum Creatinine (SCr), Urine Creatinine (UCr). | | |

**Supplementary Table S3.** Betweenness and p-values for the variables in the CardioMet12.

| Variable | Betweenness | p-values |
| --- | --- | --- |
| Abdominal obesity | N/A | N/A |
| Fatty Liver Status | 3.500 | 0.062 |
| Glycated Hemoglobin | 3.500 | 0.032 |
| LDL-C | 1.000 | 0.052 |
| Total Protein | 5.000 | 0.208 |
| Total Bilirubin | 8.000 | 0.244 |
| AKP | 3.500 | 0.138 |
| TSH | 1.910 | 0.442 |
| T4 | 17.126 | 0.494 |
| eGFR | 0.000 | 1.000 |
| Microalbumin | 6.000 | 0.314 |
| Bone density | N/A | N/A |

**Supplementary Table S4**. Variables in current models for the prediction of cardiovascular diseases (CVD).

| Model | Time | Region | Scale | Accuracy | Biomarkers |
| --- | --- | --- | --- | --- | --- |
| ASSIGN [21, 22] | 2007 | Scotland | 13,297 | 0.727-0.765 | Diabetes (β=0.81558 & 0.97727), TC (β=0.22286 & 0.12720), HDL-C (β=-0.53684 & -0.55836), age, SBP, smoking, sex, family history, SIMD score |
| Framingham [23] | 2008 | USA | 8491 | 0.763-0.793 | TC [HR=3.08 (male) & 3.35 (female), β=1.12370 (male) & 1.20904 (female)], diabetes (fasting globulin)(HR= 1.78 & 2.00, β=0.57367 & 0.69154), HDL-C (HR=0.39 & 0.49, −0.93263 & −0.70833), age, SBP, smoking |
| Globorisk [22] | 2015 | Global | 50,129 | 0.740-0.840 | diabetes (HR=2.21), TC (HR=1.18), age, SBP, smoking, sex, country |
| INTERHEART [24] | 2011 | Global | 27,043 | 0.710-0.730 | diabetes (HR=2.50), age, SBP, smoking, sex, ApoB/A1, hypertension |
| PAR [25] | 2016 | China | 21,320 | 0.794-0.811 | TC (β=0.62 & 0.06), diabetes (β=0.36 & 0.57), HDL-C (β=-0.69 & -0.22), age, SBP, smoking, WC, geographic region, urbanization, family history of ASCVD |
| PCE [26] | 2013 | USA | 24,626 | 0.746-0.806 | TC (β=11.853 & 13.540), HDL-C (β=−7.99 & -13.578), diabetes (β=0.658 & 0.661), age, SBP, smoking |
| PREDICT [27] | 2018 | New Zealand | 401,752 | 0.720-0.730 | Diabetes (HR=1.75 & 1.72), TC/HDL-C (HR=1.14 & 1.13), age, SBP, smoking, sex, NZDep quintile, ethnicity, atrial fibrillation . |
| PREVENT [28] | 2024 | USA | 3,281,919 | 0.736-0.774 | eGFR (HR= 1.61 & 1.72), diabetes (HR=1.58 & 1.65), UACR (HR= 1.21 & 1.19), HbA1c (HR= 1.11 & 1.15), non-HDL-C (HR=1.05 & 1.00), HDL-C (HR=0.92 & 0.86), age, SBP, smoking, sex, BMI, anti-hypertension medication, lipid-lowering medication, zip code |
| PROCAM [29] | 2002 | German | 5389 | 0.824-0.829 | Diabetes (HR=1.491, β=6.7), TG (HR=1.373, β=126.2), LDL-C (HR=1.013, β=148.5), HDL-C (HR=0.968, β=45.7), age, SBP, smoking, family history of MI |
| QRISK1 [30] | 2007 | England | 1,283,174 | 0.767-0.788 | TC/HDL-C (HR=1.001 & 1.001), age, SBP, smoking, sex, BMI, family history of premature cardiovascular disease, Townsend deprivation score, treated hypertension |
| QRISK2 [31] | 2008 | England | 2,285,815 | 0.792-0.817 | TC/HDL-C (HR= 1.19 & 1.17), T2D (HR=2.20 & 2.54), age, SBP, smoking, sex, BMI, family history of coronary heart disease in first degree relative under 60 years, Townsend deprivation score, ethnicity, treated hypertension, renal disease, atrial fibrillation, rheumatoid arthritis. |
| QRISK3 [32] | 2017 | England | 7,889,803 | 0.858-0.880 | Diabetes (T1D HR=3.59 & 5.66, T2D HR=2.42 & 2.95), TC/HDL-C (HR= 1.19 & 1.17), age, SBP, smoking, sex, BMI, family history of coronary heart disease, ethnic origin, treated hypertension, chronic kidney disease, atrial fibrillation, rheumatoid arthritis, migraine, corticosteroid use, SLE, atypical antipsychotic use, severe mental illness, erectile dysfunction or treatment, standard deviation of systolic blood pressure |
| Reynolds [33, 34] | 2007 | USA | 35,282 | 0.700-0.808 | TC (β=1.382 & 0.963), HbA1c [β=0.134 (female)], HDL-C (β=-1.172 & -0.772), age, SBP, smoking, sex, hsCRP, family history |
| SCORE [35] | 2003 | Europe | 205,178 | 0.670-0.820 | Age, SBP, smoking, sex, TC, HDL-C, TC/HDL, region |
| SCORE2 [36] | 2021 | Europe | 677,684 | 0.665-0.813 | Age, SBP, smoking, sex, NonHDL-C, region |
| SCORE2-OP [37] | 2021 | Europe | 28,503 | 0.650-0.660 | TC (HR=1.10 & 1.06, β=0.085 & 0.06), HDL-C (HR=0.71 & 0.75, β=-0.356 & -0.304), age, SBP, smoking, sex, region |
| WHO CVD risk [38] | 2019 | Global | 376,177 | 0.685-0.833 | Age, SBP, smoking, sex, TC, diabetes, region |

**Supplementary Table S5**. Variables in diagnostic criteria for MetS.

| Diagnosis | Time | Variables | | | | | |
| --- | --- | --- | --- | --- | --- | --- | --- |
| WHO [39] | 1998 | WC | TG | HDL-C | SBP | FBG or 2h glucose |  |
| EGIR [40] | 1999 | WC | TG | HDL-C | SBP | FBG or 2h glucose |  |
| NCEP ATPⅢ [41] | 2001 | WC | TG | HDL-C | SBP | FBG |  |
| IDF [42] | 2005 | WC | TG | HDL-C | SBP | FBG |  |
| AHA/NHLBI [43] | 2009 | WC | TG | HDL-C | SBP | FBG |  |

**Supplementary Table S6.** Bootstrap internal validation and optimism corrected performance of CardioMet12.

**Panel A. *Summary of optimism-corrected performance***

| **Metric** | **Apparent performance** | **Mean optimism** | **Optimism-corrected performance** |
| --- | --- | --- | --- |
| AUC | 0.771 | 0.013 | 0.758 |
| Brier score | 0.156 | -0.002 | 0.158 |
| Calibration slope | 1.000 | 0.063 | 0.937 |

**Panel B. *Bootstrap iteration-level optimism estimates***

| **Bootstrap ID** | **Optimism AUC** | **Optimism brier** | **Optimism calibration slope** |
| --- | --- | --- | --- |
| 1 | 0.024606152 | -0.006412639 | 0.114594799 |
| 2 | 0.015826455 | -0.002103533 | 0.094619020 |
| 3 | 0.001960235 | 0.003711468 | 0.021017379 |
| 4 | 0.015142049 | -0.004458934 | 0.057345275 |
| 5 | 0.008911880 | 0.001022028 | 0.042398366 |
| 6 | 0.015541193 | -0.002971156 | 0.056864383 |
| 7 | 0.015025641 | -0.006013523 | 0.087529602 |
| 8 | 0.009634826 | -0.001577555 | 0.053114111 |
| 9 | 0.016145349 | 0.000344662 | 0.074129325 |
| 10 | 0.009756102 | -0.003068643 | 0.045012507 |
| 11 | 0.017084287 | -0.001228386 | 0.075376185 |
| 12 | 0.002938343 | 0.001548107 | 0.036460578 |
| 13 | 0.019417453 | -0.005306648 | 0.077036275 |
| 14 | 0.024905562 | -0.002173859 | 0.096406187 |
| 15 | -0.013273316 | 0.007170132 | -0.112361085 |
| 16 | 0.004483366 | 0.002052398 | 0.016681659 |
| 17 | -0.003669636 | 0.005547492 | -0.018841622 |
| 18 | 0.018495127 | -0.005642637 | 0.097555895 |
| 19 | 0.023640243 | -0.006573525 | 0.120076174 |
| 20 | 0.018554857 | -0.005510719 | 0.080878232 |
| 21 | 0.033111391 | -0.006210526 | 0.156986149 |
| 22 | 0.010545359 | -0.001624211 | 0.075005780 |
| 23 | 0.024900124 | -0.003094259 | 0.119740901 |
| 24 | 0.019891785 | -0.004268214 | 0.098560549 |
| 25 | -0.000820763 | -0.000807982 | -0.027773095 |
| 26 | 0.002416660 | 0.002391772 | -0.002522607 |
| 27 | 0.001807183 | -0.003855592 | 0.001528792 |
| 28 | 0.013681946 | -0.000623652 | 0.066030398 |
| 29 | 0.010423850 | 0.001722718 | 0.027896276 |
| 30 | 0.025225768 | -0.002428930 | 0.120562454 |
| 31 | 0.013046539 | -0.000361558 | 0.070787246 |
| 32 | 0.011258754 | -0.004966666 | 0.054669443 |
| 33 | 0.005302518 | 0.001188901 | 0.007030759 |
| 34 | 0.014138262 | 0.000681541 | 0.050851931 |
| 35 | 0.011958496 | -0.005247998 | 0.046031859 |
| 36 | -0.003005850 | 0.004751110 | -0.009359905 |
| 37 | 0.007103796 | -0.000048004 | 0.051914162 |
| 38 | 0.015800757 | -0.002026985 | 0.094113789 |
| 39 | 0.015581972 | -0.002586584 | 0.074598089 |
| 40 | 0.029738637 | -0.005727310 | 0.162547242 |
| 41 | 0.015782181 | -0.006138619 | 0.076485921 |
| 42 | 0.011017743 | -0.001693084 | 0.084399275 |
| 43 | 0.006102139 | 0.001603371 | 0.027845926 |
| 44 | 0.011007240 | -0.000716252 | 0.051698187 |
| 45 | 0.024769344 | -0.008792713 | 0.134171053 |
| 46 | 0.006569926 | -0.005485956 | 0.037685878 |
| 47 | 0.007509849 | -0.000112539 | 0.043592553 |
| 48 | 0.018929403 | -0.002607639 | 0.104530151 |
| 49 | 0.005878211 | -0.001210561 | 0.015683057 |
| 50 | 0.014686438 | -0.001281306 | 0.084083750 |
| 51 | 0.004610787 | 0.001132609 | 0.012433848 |
| 52 | 0.015894752 | -0.004003055 | 0.087527504 |
| 53 | 0.009975781 | 0.000404769 | 0.038459699 |
| 54 | 0.011343014 | -0.005077814 | 0.064954056 |
| 55 | 0.009700541 | -0.004769957 | 0.036466440 |
| 56 | 0.017777422 | -0.004651219 | 0.088735172 |
| 57 | 0.025543568 | -0.004942691 | 0.115146613 |
| 58 | 0.012539270 | -0.001020326 | 0.058136145 |
| 59 | 0.017041717 | -0.007148627 | 0.084356370 |
| 60 | 0.022972415 | -0.004001183 | 0.098095531 |
| 61 | 0.014863047 | -0.007131459 | 0.064372019 |
| 62 | 0.012687355 | -0.005274809 | 0.065051245 |
| 63 | 0.017401501 | 0.000660365 | 0.081556924 |
| 64 | 0.007691451 | -0.000128769 | 0.045214842 |
| 65 | 0.003680008 | -0.003190809 | 0.041970670 |
| 66 | -0.001067116 | -0.003150456 | -0.002341338 |
| 67 | -0.000659773 | -0.002362096 | 0.030379774 |
| 68 | 0.015903763 | -0.005282697 | 0.083804366 |
| 69 | 0.030008781 | -0.001741609 | 0.144761423 |
| 70 | 0.017590952 | -0.005614097 | 0.061370422 |
| 71 | 0.012713788 | 0.001445219 | 0.083479430 |
| 72 | 0.021554272 | -0.004081788 | 0.114816656 |
| 73 | -0.002704930 | -0.002511116 | -0.009905110 |
| 74 | 0.014102623 | -0.003315579 | 0.073273498 |
| 75 | 0.007150152 | 0.002308388 | 0.026415692 |
| 76 | -0.004440247 | 0.002600939 | -0.023412762 |
| 77 | 0.011131244 | -0.004941637 | 0.083840581 |
| 78 | 0.015784657 | -0.001747656 | 0.071708634 |
| 79 | 0.032149828 | -0.006988360 | 0.139630649 |
| 80 | 0.010882855 | 0.001994864 | 0.032614905 |
| 81 | 0.013153865 | -0.000559125 | 0.070405886 |
| 82 | 0.030019905 | -0.006876936 | 0.147388061 |
| 83 | 0.009205448 | -0.005643270 | 0.071070113 |
| 84 | 0.004513466 | -0.010758106 | 0.042008447 |
| 85 | 0.009025423 | -0.003931281 | 0.047157793 |
| 86 | 0.019867793 | -0.003514887 | 0.089599816 |
| 87 | 0.020615338 | -0.007137032 | 0.112663841 |
| 88 | 0.009736493 | -0.002884605 | 0.052389211 |
| 89 | 0.021281166 | -0.004412213 | 0.082865642 |
| 90 | 0.008260866 | -0.002036811 | 0.014666911 |
| 91 | 0.015124648 | -0.003771293 | 0.047026385 |
| 92 | 0.004436494 | -0.002699581 | 0.014353634 |
| 93 | 0.023281018 | -0.001617358 | 0.113902331 |
| 94 | 0.010776476 | -0.000656950 | 0.050984242 |
| 95 | 0.010692949 | 0.001138237 | 0.039206326 |
| 96 | 0.019471039 | -0.005777770 | 0.104643037 |
| 97 | 0.011466837 | -0.003738894 | 0.071437457 |
| 98 | -0.004302793 | 0.001281521 | -0.003515359 |
| 99 | 0.008177569 | 0.004701947 | 0.035949216 |
| 100 | 0.021412181 | -0.005312007 | 0.106646475 |
| 101 | 0.008571505 | -0.003410027 | 0.071699662 |
| 102 | -0.005207653 | -0.002204816 | -0.028175901 |
| 103 | -0.002249233 | 0.002057634 | -0.014659549 |
| 104 | 0.008436854 | -0.001015960 | 0.018597487 |
| 105 | -0.001699007 | 0.003586205 | -0.003246854 |
| 106 | 0.004039609 | 0.003078584 | 0.038800694 |
| 107 | 0.005043760 | -0.002011609 | 0.036580872 |
| 108 | 0.013470318 | -0.001490668 | 0.073643355 |
| 109 | 0.027186399 | -0.001511784 | 0.134901956 |
| 110 | 0.027391507 | -0.001628631 | 0.111363905 |
| 111 | 0.005664777 | -0.005451252 | 0.030688597 |
| 112 | 0.014223151 | -0.004143521 | 0.079557244 |
| 113 | 0.025304754 | -0.007577217 | 0.148259185 |
| 114 | 0.029213365 | -0.012271260 | 0.123506148 |
| 115 | -0.000652668 | 0.001023571 | -0.000860093 |
| 116 | 0.021746331 | -0.002610663 | 0.119806189 |
| 117 | 0.005871591 | 0.003811806 | 0.036114571 |
| 118 | 0.010979374 | -0.005347736 | 0.029157847 |
| 119 | 0.000453916 | -0.003998804 | 0.069156567 |
| 120 | 0.008707035 | -0.001065005 | 0.015003317 |
| 121 | 0.004454991 | -0.002820560 | 0.045778470 |
| 122 | 0.018610009 | -0.005976363 | 0.096304392 |
| 123 | 0.020716951 | -0.001822097 | 0.093195158 |
| 124 | 0.021493940 | -0.004206313 | 0.112091886 |
| 125 | 0.018677030 | -0.006529517 | 0.061863877 |
| 126 | 0.018021340 | -0.002099586 | 0.115009377 |
| 127 | 0.001496938 | -0.004072785 | 0.014535907 |
| 128 | 0.015432499 | -0.006103696 | 0.059346233 |
| 129 | 0.011152932 | -0.007082629 | 0.072859526 |
| 130 | 0.014764828 | -0.000936383 | 0.064203120 |
| 131 | 0.031846550 | -0.001646025 | 0.151911138 |
| 132 | 0.020106413 | -0.007599976 | 0.095861131 |
| 133 | 0.007436011 | -0.002391279 | 0.038020433 |
| 134 | 0.006350807 | 0.000327702 | -0.002180993 |
| 135 | 0.032495160 | -0.006768055 | 0.139224927 |
| 136 | 0.015639693 | -0.000817480 | 0.060280984 |
| 137 | 0.025191096 | -0.004376983 | 0.058291926 |
| 138 | 0.018090114 | -0.002084898 | 0.098719115 |
| 139 | 0.015538859 | -0.002972369 | 0.080235917 |
| 140 | 0.006967185 | -0.001642570 | 0.022809167 |
| 141 | 0.023178416 | -0.003707162 | 0.110446086 |
| 142 | 0.015317384 | -0.003884729 | 0.091793989 |
| 143 | 0.009664501 | -0.005491936 | 0.055677381 |
| 144 | 0.017934289 | -0.008187399 | 0.086817815 |
| 145 | 0.006928077 | -0.002545018 | 0.047775771 |
| 146 | 0.029073882 | -0.007719657 | 0.128053997 |
| 147 | 0.014616534 | 0.000844936 | 0.078442765 |
| 148 | 0.011838552 | -0.005471348 | 0.051663309 |
| 149 | 0.011691166 | 0.000973716 | 0.030146149 |
| 150 | 0.023404409 | -0.005754831 | 0.111218562 |
| 151 | 0.006592510 | -0.004234112 | 0.060537762 |
| 152 | 0.018612845 | -0.001790126 | 0.096101972 |
| 153 | 0.011552265 | -0.004088040 | 0.059557502 |
| 154 | 0.003545790 | 0.001329697 | 0.020187428 |
| 155 | 0.022865650 | -0.009918454 | 0.092278840 |
| 156 | 0.004052819 | 0.001073316 | 0.032466226 |
| 157 | 0.015133453 | -0.006562244 | 0.088237021 |
| 158 | 0.033522690 | -0.008558588 | 0.144161074 |
| 159 | -0.002214573 | -0.001583148 | -0.018798086 |
| 160 | 0.021449491 | -0.000244085 | 0.094926406 |
| 161 | 0.015784372 | 0.000556266 | 0.072944529 |
| 162 | 0.014266505 | -0.002549488 | 0.074658019 |
| 163 | 0.004425920 | -0.008230356 | 0.038727123 |
| 164 | 0.022332527 | -0.004089892 | 0.104879590 |
| 165 | 0.014601004 | 0.000028285 | 0.083742032 |
| 166 | 0.014505616 | -0.002331141 | 0.095046662 |
| 167 | 0.023833507 | -0.007928776 | 0.092971100 |
| 168 | 0.017691497 | -0.006128417 | 0.098722887 |
| 169 | 0.027966161 | -0.004240894 | 0.137401572 |
| 170 | 0.013578932 | -0.000714167 | 0.072415144 |
| 171 | -0.006752393 | 0.000723759 | -0.029262973 |
| 172 | 0.022856807 | -0.010331552 | 0.111340345 |
| 173 | 0.017663284 | -0.004285936 | 0.092901986 |
| 174 | 0.006086677 | 0.001045360 | 0.048778986 |
| 175 | 0.012464344 | 0.001091166 | 0.038006425 |
| 176 | 0.015947494 | -0.005773533 | 0.098475285 |
| 177 | 0.015955859 | -0.006345367 | 0.093126583 |
| 178 | 0.020412628 | -0.005512083 | 0.063737895 |
| 179 | 0.024580123 | -0.002817757 | 0.114503309 |
| 180 | 0.006107544 | 0.000833912 | 0.032730577 |
| 181 | 0.011641675 | -0.000422543 | 0.078278327 |
| 182 | 0.011703268 | -0.000082825 | 0.089329249 |
| 183 | 0.015087598 | -0.001073486 | 0.077961673 |
| 184 | 0.014124434 | 0.000165286 | 0.069322389 |
| 185 | 0.010878670 | 0.002021605 | 0.045094163 |
| 186 | 0.014874613 | -0.004317274 | 0.073808535 |
| 187 | 0.009138122 | -0.002069012 | 0.044075567 |
| 188 | -0.009280935 | 0.002887118 | -0.024642643 |
| 189 | 0.000347641 | 0.001993120 | 0.017201970 |
| 190 | -0.006100529 | 0.005721544 | 0.020258560 |
| 191 | 0.015199665 | 0.000839861 | 0.051636671 |
| 192 | -0.000270806 | 0.003757565 | -0.040105377 |
| 193 | 0.001121367 | -0.001100958 | -0.000630623 |
| 194 | 0.017077144 | -0.003147577 | 0.106489781 |
| 195 | 0.008822487 | -0.002504454 | 0.032988747 |
| 196 | 0.002620457 | -0.002854575 | 0.018474072 |
| 197 | 0.027375735 | -0.002361813 | 0.129670946 |
| 198 | 0.006740591 | 0.001854521 | 0.025509956 |
| 199 | 0.010569967 | -0.003276646 | 0.083361963 |
| 200 | -0.010162673 | 0.004099353 | -0.095545101 |

**Supplementary Table S7.** Calibration metrics and operating thresholds for CardioMet12 models in STONE population (n = 7,11).

***Panel A. Calibration metrics***

| **Metric** | **CardioMet12** | **CardioMet12 + Age + Sex** |
| --- | --- | --- |
| Calibration intercept | -0.2331 | -0.0090 |
| Calibration slope | 0.9222 | 1.1019 |
| Brier score | 0.1400 | 0.1081 |
| Brier score (null model) | 0.1645 | 0.1645 |
| Hosmer–Lemeshow *P* | 0.0715 | 0.5302 |

***Panel B. Operating thresholds***

| **Threshold criterion** | **Cutpoint** | **Sensitivity** | **Specificity** | **PPV** | **NPV** |
| --- | --- | --- | --- | --- | --- |
| ***CardioMet12 (probability)*** |  |  |  |  |  |
| Youden optimal | 0.25 | 0.756 | 0.732 | 0.425 | 0.920 |
| High sensitivity (≥90%) | 0.09 | 0.919 | 0.393 | 0.284 | 0.949 |
| High specificity (≥90%) | 0.43 | 0.263 | 0.918 | 0.457 | 0.826 |
| ***CardioMet12 + Age + Sex (probability)*** |  |  |  |  |  |
| Youden optimal | 0.20 | 0.881 | 0.764 | 0.495 | 0.961 |
| High sensitivity (≥90%) | 0.18 | 0.900 | 0.728 | 0.465 | 0.965 |
| High specificity (≥90%) | 0.46 | 0.594 | 0.900 | 0.609 | 0.894 |

**Supplementary Table S8**. Influence of **cardiovascular-kidney-metabolic** (CKM) staging on total points.

| **Variables** | **Estimate** | **Std.Error** | **t value** | **p-value** |
| --- | --- | --- | --- | --- |
| Intercept | 29.659 | 0.381 | 77.844 | <0.001 |
| CKM stage 1 | 6.073 | 0.671 | 9.049 | <0.001 |
| CKM stage 2 | 8.839 | 0.461 | 19.184 | <0.001 |
| CKM stage 3-4 | 18.109 | 0.523 | 34.611 | <0.001 |

**Supplementary Table S9**. Assessment of 12 network indicators in CardioMet12, NHANES 2007-2012.

| **Variable** | **Label** | **English Text** |
| --- | --- | --- |
| LBXSAPSI | Alkaline phosphatase (U/L) | Alkaline phosphatase (U/L) |
| LBDSTPSI | Total protein (g/L) | Total protein (g/L) |
| LBDLDLSI | LDL-cholesterol (mmol/L) | LDL-cholesterol (mmol/L) |
| LBXGH | Glycohemoglobin (%) | Glycohemoglobin (%) |
| LBDTSH1S | Thyroid-stimulating hormone (mIU/L) | Thyroid-stimulating hormone (mIU/L) |
| LBDSTBSI | Total bilirubin (umol/L) | Total bilirubin (umol/L) |
| LBXTT4 | Thyroxine, total (T4) (ug/dL) | Thyroxine, total (T4) (ug/dL) |
| URXUMS | Albumin, urine (mg/L) | Albumin, urine (mg/L) |
| URXUCR | Creatinine, urine (mg/dL) | Creatinine, urine (mg/dL) |
| LBXTR | Triglyceride (mg/dL) | Triglyceride (mg/dL) |
| LBXSGTSI | Gamma glutamyl transferase (U/L) | Gamma glutamyl transferase (U/L) |
| BMXWAIST | Waist Circumference (cm) | Waist Circumference (cm) |
| BMXWT | Weight (kg) | Weight (kg) |
| DXXOFBMD | Total femur BMD (g/cm²) | Total femur bone mineral density (g/cm²) |
| DXXNKBMD | Femoral neck BMD (g/cm²) | Femoral neck bone mineral density (g/cm²) |
| DXXTRBMD | Trochanter BMD (g/cm²) | Trochanter bone mineral density (g/cm²) |
| DXXL1BMD | L1 BMD (g/cm²) | L1 bone mineral density (g/cm²) |
| DXXL2BMD | L2 BMD (g/cm²) | L2 bone mineral density (g/cm²) |
| DXXL3BMD | L3 BMD (g/cm²) | L3 bone mineral density (g/cm²) |
| DXXL4BMD | L4 BMD (g/cm²) | L4 bone mineral density (g/cm²) |
| MCQ160c | Ever told you had coronary heart disease | Has a doctor or other health professional ever told {you/SP} that {you/s/he} . . .had coronary heart disease? |
| MCQ160b | Ever told had congestive heart failure | Has a doctor or other health professional ever told {you/SP} that {you/s/he} . . .had congestive heart failure? |
| MCQ160e | Ever told you had heart attack | Has a doctor or other health professional ever told {you/SP} that {you/s/he} . . .had a heart attack (also called myocardial infarction)? |
| MCQ160f | Ever told you had a stroke | Has a doctor or other health professional ever told {you/SP} that {you/s/he} . . .had a stroke? |
| MCQ160d | Ever told you had angina/angina pectoris | Has a doctor or other health professional ever told {you/SP} that {you/s/he} . . .had angina, also called angina pectoris? |

**Supplementary Table S10**. Baseline characteristics of U.S. adults (n = 1620) from NHANES 2007-2012, including profiles of 12 related biomarkers in CardioMet12, stratified by the presence or absence of CVD.

| **Characteristic ^A^** | **CVD (n = 119)** | **Without CVD (n = 1,501)** | **p-value** |
| --- | --- | --- | --- |
| Sex \| \| \| 0.176; Female \| 42.3 (51) \| 50.0 (767) \|; Male \| 57.7 (68) \| 50.0 (734) \|; Age (years) \| 63 (52, 72) \| 41 (30, 52) \| <0.001; Abdominal obesity \| \| \| 0.003; No \| 32.4 (38) \| 53.8 (762) \|; Yes \| 67.6 (81) \| 46.2 (739) \|; HbA1c (%) \| 5.7 (5.5, 6.4) \| 5.3 (5.1, 5.6) \| <0.001; Fatty liver disease \| \| \| 0.007; No \| 44.5 (52) \| 63.0 (920) \|; Yes \| 55.5 (67) \| 37.0 (581) \|; LDL-C (mmol/L) \| 2.51 (1.97, 3.16) \| 2.95 (2.43, 3.54) \| 0.002; Total bilirubin (µmol/L) \| 13.68 (11.97, 17.10) \| 13.68 (10.26, 17.10) \| 0.037; Total protein (g/L) \| 72 (69, 74) \| 71 (69, 74) \| 0.915; ALP (U/L) \| 67 (57, 85) \| 62 (51, 75) \| <0.001; eGFR (mL/min/1.73 m²) \| 85.04 (71.83, 100.69) \| 102.67 (90.66, 114.27) \| <0.001; 24-h urine microalbumin (g/L) \| 11.55 (7.10, 23.76) \| 7.88 (5.55, 12.85) \| <0.001; T4 (nmol/L) \| 101.67 (87.52, 118.40) \| 97.81 (86.23, 111.97) \| 0.079; TSH (mIU/L) \| 1.57 (0.98, 2.58) \| 1.67 (1.13, 2.45) \| 0.937; Osteoporosis \| \| \| 0.002; No \| 88.6 (102) \| 95.3 (1,402) \|; Yes \| 11.4 (17) \| 4.7 (99) \| ^A^ Data are presented as weighted median (Q1, Q3) for continuous variables and weighted percentage (unweighted n) for categorical variables. | | | |

**Supplementary Table S11.** Comparison of baseline characteristics between included and excluded participants in the NHANES 2007-2012 cohort.

| **Variable** | **Included (*n* = 1,620)** | **Excluded (*n* = 16,093)** | ***P*** |
| --- | --- | --- | --- |
| *Continuous variables, median (Q1, Q3)* |  |  |  |
| Age, years | 45.0 (33.0, 59.0) | 50.0 (35.0, 65.0) | <0.001 |
| HbA1c, % | 5.4 (5.2, 5.7) | 5.5 (5.3, 5.9) | <0.001 |
| LDL-C, mmol/L | 2.9 (2.4, 3.5) | 2.9 (2.3, 3.5) | 0.204 |
| Total bilirubin, μmol/L | 13.7 (10.3, 17.1) | 12.0 (10.3, 15.4) | <0.001 |
| Total protein, g/L | 72.0 (69.0, 75.0) | 72.0 (69.0, 75.0) | 0.050 |
| ALP, U/L | 65.0 (53.0, 79.0) | 66.0 (54.0, 81.0) | 0.015 |
| TSH, mIU/L | 1.6 (1.1, 2.5) | 1.6 (1.0, 2.3) | 0.012 |
| T4, nmol/L | 99.1 (87.5, 113.3) | 100.4 (88.8, 114.2) | 0.130 |
| eGFR, mL/min/1.73 m² | 100.0 (85.8, 113.3) | 95.6 (78.2, 111.2) | <0.001 |
| *Categorical variables* |  |  |  |
| Male sex, % | 49.5 | 48.8 | 0.608 |
| Prevalent CVD, % | 7.3 | 11.9 | <0.001 |

**Supplementary Table S12**. Influence of cardiovascular-kidney-metabolic (CKM) staging on CardioMet12 total points in the NHANES validation cohort (n = 1,620).

| **CKM Stage** | ***n*** | **Weighted Mean** | **SE** | **β** | **95% CI** | ***P*** |
| --- | --- | --- | --- | --- | --- | --- |
| Stage 0 (ref) | 197 | 33.87 | 0.89 | — | — | — |
| Stage 1 | 486 | 41.29 | 0.55 | 7.42 | 5.45, 9.40 | <0.001 |
| Stage 2 | 753 | 45.59 | 0.76 | 11.73 | 9.39, 14.06 | <0.001 |
| Stage 3–4 | 184 | 53.09 | 0.84 | 19.22 | 16.30, 22.15 | <0.001 |

**Supplementary Table S13.** Comparative Performance of CardioMet12, Framingham, and PREVENT in NHANES population.

| **Model** | **AUC** | **Sensitivity** | **Specificity** |
| --- | --- | --- | --- |
| CardioMet12 | 0.723 | 0.882 | 0.465 |
| CardioMet12_E_ | 0.827 | 0.714 | 0.809 |
| Framingham (general) | 0.819 | 0.824 | 0.718 |
| Framingham (-age-sex) | 0.630 | 0.613 | 0.580 |
| PREVENT | 0.841 | 0.756 | 0.797 |

**Supplementary Table S14.** Calibration metrics and operating thresholds for CardioMet12 models in NHANES population (n = 1,620).

***Panel A. Calibration metrics***

| **Metric** | **CardioMet12 (raw)** | **CardioMet12 (recalibrated)** | **CardioMet12 + Age + Sex** |
| --- | --- | --- | --- |
| Calibration intercept | -2.073 | -0.401 | 0.000 |
| Calibration slope | 0.817 | 0.817 | 1.000 |
| Brier score | 0.148 | 0.0646 | 0.0591 |
| Brier score (null model) | 0.0681 | 0.0681 | 0.0681 |
| Hosmer–Lemeshow *P* | ＜0.001 | 0.049 | 0.595 |

***Panel B. Operating thresholds***

| **Threshold criterion** | **Cutpoint** | **Sensitivity** | **Specificity** | **PPV** | **NPV** |
| --- | --- | --- | --- | --- | --- |
| ***CardioMet12 (raw score)*** |  |  |  |  |  |
| Youden optimal | 43.5 | 0.882 | 0.464 | 0.116 | 0.980 |
| High sensitivity (≥90%) | 39.5 | 0.908 | 0.380 | 0.104 | 0.981 |
| High specificity (≥90%) | 59.5 | 0.303 | 0.918 | 0.226 | 0.943 |
| ***CardioMet12 + Age + Sex (probability)*** |  |  |  |  |  |
| Youden optimal | 0.10 | 0.714 | 0.809 | 0.228 | 0.973 |
| High sensitivity (≥90%) | 0.04 | 0.908 | 0.555 | 0.139 | 0.987 |
| High specificity (≥90%) | 0.20 | 0.513 | 0.901 | 0.290 | 0.959 |

**Supplementary Table S15.** Comparison of survey-weighted and unweighted AUC for CVD discrimination models in the NHANES validation cohort (n = 1,620).

| **Model** | **Weighted AUC** | **Unweighted AUC (95% CI)** | **Unweighted 95% CI** | **Δ** |
| --- | --- | --- | --- | --- |
| CardioMet12 | 0.727 | 0.722 | 0.679–0.766 | +0.004 |
| CardioMet12 + Age + Sex | 0.834 | 0.827 | 0.790–0.864 | +0.007 |
| Framingham (general CVD) | 0.832 | 0.819 | 0.784–0.854 | +0.013 |
| Framingham (no age/sex) | 0.619 | 0.630 | 0.576–0.684 | −0.011 |
| PREVENT | 0.851 | 0.841 | 0.806–0.876 | +0.010 |

**Supplementary Table S16.** Subgroup analysis of CardioMet12 discrimination performance by sex and age in the validation set of STONE population (n = 771). CM12_E_: In the sex-stratified subgroup, sex was intentionally excluded from the CM12_E_ model to avoid circularity and ensure independent validation of model performance within each sex category.

| **Subgroup** | ***n*** | **CVD** | **CardioMet12 AUC (95% CI)** | **CM12_E_ AUC (95% CI)** | ***P* interaction** |
| --- | --- | --- | --- | --- | --- |
| ***Sex*** |  |  |  |  |  |
| Female | 439 | 76 | 0.778 (0.721-0.834) | 0.873 (0.837-0.909) | 0.872 |
| Male | 332 | 84 | 0.748 (0.691-0.805) | 0.889 (0.850-0.928) |  |
| ***Age*** |  |  |  |  |  |
| <41 years | 392 | 8 | 0.562 (0.305-0.819) | 0.708 (0.595-0.822) | 0.833 |
| ≥41 years | 379 | 152 | 0.631 (0.574-0.687) | 0.747 (0.697-0.796) |  |

**Supplementary Table S17.** Subgroup analysis of CardioMet12 discrimination performance by sex, age, and race/ethnicity in the NHANES validation cohort (n = 1,620).

| **Subgroup** | ***n*** | **CVD** | **CardioMet12 AUC (95% CI)** | **CM12_E_ AUC (95% CI)** | ***P* interaction** |
| --- | --- | --- | --- | --- | --- |
| ***Sex*** |  |  |  |  |  |
| Female | 818 | 51 | 0.720 (0.654–0.786) | 0.809 (0.746–0.871) | 0.649 |
| Male | 802 | 68 | 0.729 (0.671–0.787) | 0.839 (0.795–0.882) |  |
| ***Age*** |  |  |  |  |  |
| <45 years | 784 | 10 | 0.509 (0.325–0.693) | 0.615 (0.422–0.808) | 0.003 |
| ≥45 years | 836 | 109 | 0.603 (0.546–0.660) | 0.738 (0.687–0.788) |  |
| ***Race/Ethnicity*** |  |  |  |  |  |
| Non-Hispanic White | 798 | 66 | 0.710 (0.646–0.775) | 0.814 (0.763–0.866) | 0.078 |
| Non-Hispanic Black | 282 | 22 | 0.692 (0.590–0.794) | 0.812 (0.719–0.905) |  |
| Other | 540 | 31 | 0.751 (0.675–0.827) | 0.859 (0.792–0.925) |  |

**Supplementary Table S18**. Reagents and reference intervals for indicators.

| **Inspection items** | **Reagent** | **Biomarkers** | **Reference intervals** |
| --- | --- | --- | --- |
| Blood routine | NF-3E1000  NF-3W1000  DS DILUENT  M-68PLD  M-68PLN  M-68PDR  M-68PLH  M-68PFD | Red blood cell count (RBC) | 10^12^/L |
|  |  | White blood cell count (WBC) | 4.0-10.0×10^9^/L |
|  |  | Platelet count (PLT) | 10^9^/L |
| Liver function |  | Total bilirubin (TBIL) | 1.71-17.1 μmol/L |
|  |  | Direct bilirubin (DBIL) | 1.71-7 μmol/L |
|  | Total protein Test Kit | Total protein (TP) | 60-80 g/L |
|  |  | Albumin (ALB) | 40-55 g/L |
|  | ASTL | Aspartate aminotransferase (AST) | 0-40 U/L (m), 0-32 U/L (f) |
|  | ALTL | Alanine aminotransferase (ALT) | 0-41 U/L (m), 0-33 U/L (f) |
|  | GGT-2 | Gamma-glutamyltransferase (GGT) | 0-60 U/L (m), 0-40 U/L (f) |
|  | ALP2 | Alkaline phosphatase (ALP) | 40-130 U/L (m), 35-105 U/L (f) |
| Renal function | CREP2 | Serum creatinine (SCr) | 45-104 μmol/L |
|  | UA2 | Serum urea | 2.78-8.07 mmol/L |
| Electrolytes |  | Kalium (K) | 3.5-5.5 mmol/L |
|  |  | Sodium (Na) | 135-145 mmol/L |
|  |  | Chloride (Cl) | 95-105 mmol/L |
|  |  | Calcium (Ca) | 2.25-2.58 mmol/L |
| Lipid profile | CHOD-PAP, CHOL2 | Total cholesterol (TC) | 0-5.17 mmol/L |
|  | TRIGL, GPO-PAP | Triglycerides (TG) | 0-2.26 mmol/L, 0-2.30 mmol/L |
|  | HDLC4 | High-density lipoprotein cholesterol (HDL-C) | 1.04-1.55 mmol/L |
|  | LDLC3 | Low-density lipoprotein cholesterol (LDL-C) | 0-2.59 mmol/L |
|  | LPA2 | LP (a) | 20-40 mg/dL |
|  | APOAT | ApoA1 | 0.10-3.05 g/L |
|  | APOBT | ApoB | 0.60-1.10 g/L |
| Immunological examination |  | Erythrocyte sedimentation rate (ESR) | 0-20 mm/h |
|  | C-Reactive Protein (immunoturbidimetry) | C-reactive protein (CRP) | 0-8 mg/L |
|  | C3C-2 | C3 | 0.80-1.60 g/L |
|  | C4-2 | C4 | 0.1-0.4 g/L |
| Thyroid function | Triiodothyronine Assay Kit | Triiodothyronine (T3) | 0.75-2.0 ng/mL |
|  | Thyroxine Assay Kit | Tetraiodothyronine (T4) | 4.5-13.5 μg/dL |
|  | FT3 | Free triiodothyronine (FT3) | 3.5-6.5 pmol/L |
|  | FT4 | Free tetraiodothyronine (FT4) | 11.5-22.7 pmol/L |
|  | TSH3-Ultro (TSH3-UL) | Thyrotropin (TSH) | 0.55-4.78 mIU/L |
|  |  | Thyroglobulin (TG) | 3-40 μg/L |
|  | CLIA | Thyroglobulin antibody (TGAb) | 0-115 IU/ml |
|  | CLIA | Thyrotropin receptor antibody (TRAb) | 0-1.90 IU/L |
|  | CLIA | Antithyroperoxidase antibody (TPOAb) | 0-34 IU/mL |
| Glucose metabolism | Elecsys C-Peptide | Fasting insulin | 4.00-16.80 μU/ml |
|  | Elecsys C-Peptide | Fasting C-peptide | 1.10-4.40 μg/L |
|  |  | Glycated hemoglobin (HbA_1c_) | 4.0-6.0% |
|  | Glucose HK (hexokinase method), GLUC3 | Fasting glucose (FBG) | 4.4-6.1 mmol/L |
|  | Glucose HK (hexokinase method), GLUC3 | 120-minute postprandial glucose | 4.4-7.8 mmol/L |
| Urinalysis | GSB04-2834-2011 | Uric acid (UA) | 2200-5475 μmol/L |
|  | CREP2 | Urine creatinine (UCr) | 2.55-24.6 mmol/L |
|  | ALBT2 | Microalbumin | 0-30 mg/24h |
|  | UREAL (UREA/BUN) | Urinary iodine | 100-200 μg/L |

**Supplementary Table S19**. Sensitivity analyses replacing overridden indicators with each network-top alternative.

| **Sensitivity analysis** | **Variables swapped** | **AUC** | **Delta vs ref** |
| --- | --- | --- | --- |
| CardioMet12 primary | None | 0.771 | ref |
| Obesity alternative | BMI instead of abdominal obesity | 0.784 | 0.013 |
| Liver alternative (FDR-top) | ALT instead of total bilirubin | 0.784 | 0.013 |
| Liver alternative (raw-P top) | Gallbladder none instead of total bilirubin | 0.784 | 0.013 |
| Thyroid alternative (raw-P top) | TPO-Ab instead of T4 + TSH | 0.783 | 0.012 |
| Thyroid alternative (FDR-top) | TG instead of T4 + TSH | 0.780 | 0.009 |
| CKD partial-override check | Remove eGFR | 0.719 | -0.052 |

**References**

[1] Zhang C, Rexrode KM, van Dam RM, Li TY, Hu FB. Abdominal obesity and the risk of all-cause, cardiovascular, and cancer mortality: sixteen years of follow-up in US women. Circulation*.* 2008; 117: 1658-1667.

[2] Yusuf S, Hawken S, Ounpuu S, Bautista L, Franzosi MG, Commerford P*, et al.* Obesity and the risk of myocardial infarction in 27,000 participants from 52 countries: a case-control study. Lancet*.* 2005; 366: 1640-1649.

[3] Ridker PM, Moorthy MV, Cook NR, Rifai N, Lee IM, Buring JE. Inflammation, Cholesterol, Lipoprotein(a), and 30-Year Cardiovascular Outcomes in Women. N Engl J Med*.* 2024; : .

[4] Ference BA, Braunwald E, Catapano AL. The LDL cumulative exposure hypothesis: evidence and practical applications. Nat Rev Cardiol*.* 2024; 21: 701-716.

[5] Di Angelantonio E, Gao P, Khan H, Butterworth AS, Wormser D, Kaptoge S*, et al.* Glycated hemoglobin measurement and prediction of cardiovascular disease. Jama*.* 2014; 311: 1225-1233.

[6] Selvin E, Marinopoulos S, Berkenblit G, Rami T, Brancati FL, Powe NR*, et al.* Meta-analysis: glycosylated hemoglobin and cardiovascular disease in diabetes mellitus. Ann Intern Med*.* 2004; 141: 421-431.

[7] Li C, Wu W, Song Y, Xu S, Wu X. The Nonlinear Relationship Between Total Bilirubin and Coronary Heart Disease: A Dose-Response Meta-Analysis. Front Cardiovasc Med*.* 2021; 8: 761520.

[8] Ma J, Hwang SJ, Pedley A, Massaro JM, Hoffmann U, Chung RT*, et al.* Bi-directional analysis between fatty liver and cardiovascular disease risk factors. J Hepatol*.* 2017; 66: 390-397.

[9] Lee H, Lee YH, Kim SU, Kim HC. Metabolic Dysfunction-Associated Fatty Liver Disease and Incident Cardiovascular Disease Risk: A Nationwide Cohort Study. Clin Gastroenterol Hepatol*.* 2021; 19: 2138-2147.e2110.

[10] Haarhaus M, Brandenburg V, Kalantar-Zadeh K, Stenvinkel P, Magnusson P. Alkaline phosphatase: a novel treatment target for cardiovascular disease in CKD. Nat Rev Nephrol*.* 2017; 13: 429-442.

[11] Mittag J, Lyons DJ, Sällström J, Vujovic M, Dudazy-Gralla S, Warner A*, et al.* Thyroid hormone is required for hypothalamic neurons regulating cardiovascular functions. J Clin Invest*.* 2013; 123: 509-516.

[12] Sun X, Sun Y, Li WC, Chen CY, Chiu YH, Chien HY*, et al.* Association of thyroid-stimulating hormone and cardiovascular risk factors. Intern Med*.* 2015; 54: 2537-2544.

[13] Matsushita K, van der Velde M, Astor BC, Woodward M, Levey AS, de Jong PE*, et al.* Association of estimated glomerular filtration rate and albuminuria with all-cause and cardiovascular mortality in general population cohorts: a collaborative meta-analysis. Lancet*.* 2010; 375: 2073-2081.

[14] Gansevoort RT, Matsushita K, van der Velde M, Astor BC, Woodward M, Levey AS*, et al.* Lower estimated GFR and higher albuminuria are associated with adverse kidney outcomes. A collaborative meta-analysis of general and high-risk population cohorts. Kidney Int*.* 2011; 80: 93-104.

[15] Matsushita K, Coresh J, Sang Y, Chalmers J, Fox C, Guallar E*, et al.* Estimated glomerular filtration rate and albuminuria for prediction of cardiovascular outcomes: a collaborative meta-analysis of individual participant data. Lancet Diabetes Endocrinol*.* 2015; 3: 514-525.

[16] Inker LA, Eneanya ND, Coresh J, Tighiouart H, Wang D, Sang Y*, et al.* New Creatinine- and Cystatin C-Based Equations to Estimate GFR without Race. N Engl J Med*.* 2021; 385: 1737-1749.

[17] Shi J, Wu Y, Zhu S, Xie Y, Xiang M. The Association between Serum Creatinine/Cystatin C Ratio and Cardiovascular Morbidity and Mortality: Insights from NHANES. Rev Cardiovasc Med*.* 2023; 24: 275.

[18] Tankó LB, Christiansen C, Cox DA, Geiger MJ, McNabb MA, Cummings SR. Relationship between osteoporosis and cardiovascular disease in postmenopausal women. J Bone Miner Res*.* 2005; 20: 1912-1920.

[19] van den Bos F, Emmelot-Vonk MH, Verhaar HJ, van der Schouw YT. Links between Atherosclerosis and Osteoporosis in Middle Aged and Elderly Men. J Nutr Health Aging*.* 2018; 22: 639-644.

[20] Hu X, Ma S, Chen L, Tian C, Wang W. Association between osteoporosis and cardiovascular disease in elderly people: evidence from a retrospective study. PeerJ*.* 2023; 11: e16546.

[21] Woodward M, Brindle P, Tunstall-Pedoe H. Adding social deprivation and family history to cardiovascular risk assessment: the ASSIGN score from the Scottish Heart Health Extended Cohort (SHHEC). Heart*.* 2007; 93: 172-176.

[22] Hajifathalian K, Ueda P, Lu Y, Woodward M, Ahmadvand A, Aguilar-Salinas CA*, et al.* A novel risk score to predict cardiovascular disease risk in national populations (Globorisk): a pooled analysis of prospective cohorts and health examination surveys. Lancet Diabetes Endocrinol*.* 2015; 3: 339-355.

[23] D'Agostino RB, Sr., Vasan RS, Pencina MJ, Wolf PA, Cobain M, Massaro JM*, et al.* General cardiovascular risk profile for use in primary care: the Framingham Heart Study. Circulation*.* 2008; 117: 743-753.

[24] McGorrian C, Yusuf S, Islam S, Jung H, Rangarajan S, Avezum A*, et al.* Estimating modifiable coronary heart disease risk in multiple regions of the world: the INTERHEART Modifiable Risk Score. Eur Heart J*.* 2011; 32: 581-589.

[25] Yang X, Li J, Hu D, Chen J, Li Y, Huang J*, et al.* Predicting the 10-Year Risks of Atherosclerotic Cardiovascular Disease in Chinese Population: The China-PAR Project (Prediction for ASCVD Risk in China). Circulation*.* 2016; 134: 1430-1440.

[26] Goff DC, Jr., Lloyd-Jones DM, Bennett G, Coady S, D'Agostino RB, Gibbons R*, et al.* 2013 ACC/AHA guideline on the assessment of cardiovascular risk: a report of the American College of Cardiology/American Heart Association Task Force on Practice Guidelines. Circulation*.* 2014; 129: S49-73.

[27] Pylypchuk R, Wells S, Kerr A, Poppe K, Riddell T, Harwood M*, et al.* Cardiovascular disease risk prediction equations in 400 000 primary care patients in New Zealand: a derivation and validation study. Lancet*.* 2018; 391: 1897-1907.

[28] Osude N, Granger C. PREVENT equations predicted risk for incident CVD in adults aged 30 to 79 y. Ann Intern Med*.* 2024; 177: Jc71.

[29] Assmann G, Cullen P, Schulte H. Simple scoring scheme for calculating the risk of acute coronary events based on the 10-year follow-up of the prospective cardiovascular Münster (PROCAM) study. Circulation*.* 2002; 105: 310-315.

[30] Hippisley-Cox J, Coupland C, Vinogradova Y, Robson J, May M, Brindle P. Derivation and validation of QRISK, a new cardiovascular disease risk score for the United Kingdom: prospective open cohort study. Bmj*.* 2007; 335: 136.

[31] Hippisley-Cox J, Coupland C, Vinogradova Y, Robson J, Minhas R, Sheikh A*, et al.* Predicting cardiovascular risk in England and Wales: prospective derivation and validation of QRISK2. Bmj*.* 2008; 336: 1475-1482.

[32] Hippisley-Cox J, Coupland C, Brindle P. Development and validation of QRISK3 risk prediction algorithms to estimate future risk of cardiovascular disease: prospective cohort study. Bmj*.* 2017; 357: j2099.

[33] Ridker PM, Buring JE, Rifai N, Cook NR. Development and validation of improved algorithms for the assessment of global cardiovascular risk in women: the Reynolds Risk Score. Jama*.* 2007; 297: 611-619.

[34] Ridker PM, Paynter NP, Rifai N, Gaziano JM, Cook NR. C-reactive protein and parental history improve global cardiovascular risk prediction: the Reynolds Risk Score for men. Circulation*.* 2008; 118: 2243-2251, 2244p following 2251.

[35] Conroy RM, Pyörälä K, Fitzgerald AP, Sans S, Menotti A, De Backer G*, et al.* Estimation of ten-year risk of fatal cardiovascular disease in Europe: the SCORE project. Eur Heart J*.* 2003; 24: 987-1003.

[36] SCORE2 risk prediction algorithms: new models to estimate 10-year risk of cardiovascular disease in Europe. Eur Heart J*.* 2021; 42: 2439-2454.

[37] SCORE2-OP risk prediction algorithms: estimating incident cardiovascular event risk in older persons in four geographical risk regions. Eur Heart J*.* 2021; 42: 2455-2467.

[38] World Health Organization cardiovascular disease risk charts: revised models to estimate risk in 21 global regions. Lancet Glob Health*.* 2019; 7: e1332-e1345.

[39] Alberti KG, Zimmet PZ. Definition, diagnosis and classification of diabetes mellitus and its complications. Part 1: diagnosis and classification of diabetes mellitus provisional report of a WHO consultation. Diabet Med*.* 1998; 15: 539-553.

[40] Balkau B, Charles MA. Comment on the provisional report from the WHO consultation. European Group for the Study of Insulin Resistance (EGIR). Diabet Med*.* 1999; 16: 442-443.

[41] Executive Summary of The Third Report of The National Cholesterol Education Program (NCEP) Expert Panel on Detection, Evaluation, And Treatment of High Blood Cholesterol In Adults (Adult Treatment Panel III). Jama*.* 2001; 285: 2486-2497.

[42] Eckel RH, Alberti KG, Grundy SM, Zimmet PZ. The metabolic syndrome. Lancet*.* 2010; 375: 181-183.

[43] Alberti KG, Eckel RH, Grundy SM, Zimmet PZ, Cleeman JI, Donato KA*, et al.* Harmonizing the metabolic syndrome: a joint interim statement of the International Diabetes Federation Task Force on Epidemiology and Prevention; National Heart, Lung, and Blood Institute; American Heart Association; World Heart Federation; International Atherosclerosis Society; and International Association for the Study of Obesity. Circulation*.* 2009; 120: 1640-1645.
